# JAK/STAT and Hox Dynamic Interactions in an Organogenetic Gene Cascade

**DOI:** 10.1371/journal.pgen.1005412

**Published:** 2015-07-31

**Authors:** Pedro B. Pinto, Jose Manuel Espinosa-Vázquez, María Luísa Rivas, James Castelli-Gair Hombría

**Affiliations:** Centro Andaluz de Biología de Desarrollo, CSIC/JA, Universidad Pablo de Olivde, Seville, Spain; Harvard Medical School, UNITED STATES

## Abstract

Organogenesis is controlled by gene networks activated by upstream selector genes. During development the gene network is activated stepwise, with a sequential deployment of successive transcription factors and signalling molecules that modify the interaction of the elements of the network as the organ forms. Very little is known about the steps leading from the early specification of the cells that form the organ primordium to the moment when a robust gene network is in place. Here we study in detail how a Hox protein induces during early embryogenesis a simple organogenetic cascade that matures into a complex gene network through the activation of feedback and feed forward interaction loops. To address how the network organization changes during development and how the target genes integrate the genetic information it provides, we analyze in *Drosophila* the induction of posterior spiracle organogenesis by the Hox gene *Abdominal-B* (*Abd-B*). Initially, Abd-B activates in the spiracle primordium a cascade of transcription factors and signalling molecules including the JAK/STAT signalling pathway. We find that at later stages STAT activity feeds back directly into *Abd-B*, initiating the transformation of the Hox cascade into a gene-network. Focusing on *crumbs*, a spiracle downstream target gene of Abd-B, we analyze how a modular *cis* regulatory element integrates the dynamic network information set by Abd-B and the JAK/STAT signalling pathway during development. We describe how a Hox induced genetic cascade transforms into a robust gene network during organogenesis due to the repeated interaction of Abd-B and one of its targets, the JAK/STAT signalling cascade. Our results show that in this network STAT functions not just as a direct transcription factor, but also acts as a "counter-repressor", uncovering a novel mode for STAT directed transcriptional regulation.

## Author Summary

Organogenesis is controlled by gene networks activated by upstream selector genes. To address how the network organization changes during development and how the target genes integrate the genetic information it provides, we analyze in *Drosophila* the induction of posterior spiracle organogenesis by the Hox gene *Abdominal-B* (*Abd-B)*. Initially, Abd-B activates in the spiracle primordium a cascade of transcription factors and signalling molecules including the JAK/STAT pathway. We find that at later stages STAT activity feeds back into *Abd-B*, initiating the transformation of the Hox cascade into a gene-network. Focusing on a spiracle downstream target gene of Abd-B, we analyze how its *cis* regulatory elements integrate the dynamic network information set by Abd-B and the JAK/STAT signalling pathway during development. Our results also show that the well known transcription factor STAT can control gene expression as a “counter-repressor”, uncovering an alternative novel mode for STAT directed transcriptional regulation.

## Introduction

Organogenesis is controlled by the activation of complex gene regulatory networks in precise positions of the organism [1]. *Selector genes*, or *master regulator genes*, encode transcription factors required for the expression of entire organogenetic gene regulatory networks and, when expressed ectopically, can induce the formation of additional organs at new locations [2,3]. Prominent examples are Eyeless (Ey) capable of inducing ectopic eyes; or the Hox proteins capable of inducing segment specific organs [1,4]. However, these genes by themselves are unable to provide all the information required to specify an organ. For example, the organ specified by a particular Hox protein varies at different positions of the segment depending on its interaction with tissue-specific transcription factors and signalling pathway effectors active in each region. Similarly, ectopic Ey expression can only induce the formation of additional eyes at certain locations of the imaginal discs showing that the functional outcome of these proteins is locally modulated. Once a regulatory gene network is selected, each network gene has to be precisely activated in time and space and this is usually controlled through the gene’s non-coding *cis*-regulatory modules (CRMs). Thus, CRMs play a critical role since they act as integrators of specific combinations of transregulatory transcription factors and signalling pathway effectors, resulting in localized transcriptional activity.

Examples of Hox-induced organogenetic networks include the formation of the corpora allata or the maxillary cirri by Deformed (Dfd); the development of the prothoracic or the salivary glands by Sex combs reduced (Scr); or the formation of the posterior spiracles by Abdominal-B (Abd-B) [5–8]. In all these cases the Hox protein is the most upstream activator of an organogenetic gene-cascade. This simple and linear view of the role of *Hox* genes in the establishment of gene networks, contrasts with what is known for other selector genes. Eye development shows a more complex situation where Ey is not simply upstream of a linear cascade but forms part of the gene network itself, collaborating with other transcription factors as Eyes absent, Sine oculis or Dachshund that are equally important to induce eye organogenesis [9].

The formation of the posterior spiracles of *Drosophila* is an excellent model to study how a Hox protein controls organogenesis [8,10]. The posterior spiracles constitute the only external opening of the trachea when the larva hatches so their development has to be completed during embryogenesis. Posterior spiracle organogenesis is induced in the eighth abdominal (A8) segment when the Abdominal-B (Abd-B) Hox selector protein activates a series of primary targets that include the transcription factor genes *cut* (*ct*), *empty spiracles* (*ems*) and *spalt* (*sal*) as well as the *unpaired (upd)* and *upd2* genes encoding ligands for the JAK/STAT pathway receptor. Downstream of these primary targets, a number of secondary target genes are activated which require the activity of the primary targets to be expressed. Secondary targets include other transcription factors, but also genes encoding realizators of organogenesis, like non-classic cadherins involved in cell adhesion, RhoGAP and RhoGEF GTPase regulatory proteins influencing cytoskeletal organization, and cell polarity determinants [11]. Central among the cell polarity genes is *crumbs* (*crb*), one of the major determinants of apico-basal polarity [12,13]. Crb is a transmembrane protein that localizes to the subapical region of all ectodermal cells where it is required to maintain the epithelial structure by interaction with cortical proteins also involved in apico-basal polarity regulation. Although Crb is ubiquitous in all ectodermal cells, its expression is increased in the cells that invaginate to form the posterior spiracles (S1A and S1B Fig and [11]). The enhancement of *crb* transcription in the spiracle primordium is controlled by a posterior spiracle specific CRM regulated by JAK/STAT signalling (S1D and S1E Fig). In turn, JAK/STAT signalling in the posterior spiracles depends on *upd* transcription that is controlled by Abd-B (S1C and S1F Fig and [11]). Although this simple linear model, where the *crb*-spiracle enhancer expression is solely dependent on JAK/STAT activation, can explain why it is expressed in the spiracle primordia of A8 (S1C and S1D Fig), it does not explain why this enhancer is not also activated in the tracheal pits, where *upd* is transcribed and the JAK/STAT signalling cascade is also active (S1C and S1G–S1G” Fig). Thus, the regulation of the *crb* posterior spiracle CRM must be more complex to attain organ specificity.

To understand how organ specific gene expression is regulated in a Hox-induced morphogenetic cascade during organogenesis, we have studied the transcriptional activation of *crb* in the posterior spiracles. Our results show that spiracle specific expression of this downstream secondary target requires the cooperation of the primary targets and the Abd-B Hox selector protein. Moreover, we show that the simple linear cascade initiated by Abd-B early in embryogenesis, soon becomes a complex network due to feedback loops set up by the primary targets. Furthermore, we find evidence that STAT can influence gene expression in a novel way, not just as a positive transcription factor, but also as a counter-repressor. The *crb* posterior spiracle expression is controlled by an unanticipated number of interactions between modular elements of the CRM that influence the final transcriptional outcome.

## Results

### A crb518 minimal enhancer directs expression to the posterior spiracle in a JAK/STAT dependent manner

The reporter construct *crb43*.*2-lacZ* contains a 2kb *crb* intronic region driving posterior spiracle expression. It was shown that the activity of this reporter depends on activation of JAK/STAT signalling and that mutation of the STAT binding sites present in this element downregulate its activity [[11,14] and S1 Fig, compare E and F]. These initial studies suggested that the *crb43*.*2* enhancer is a direct target of the JAK/STAT pathway. To confirm this observation, we expressed activated STAT-GFP in S2 cells and performed Chromatin Immunoprecipitation (ChIP) using an anti-GFP antibody (S2A Fig). The enrichment of input recovery shows that STAT is able to bind directly to the *crb* enhancer, reinforcing the view that *crb* is a direct target of the JAK/STAT pathway.

To identify the minimal sequence requirements for *crb* posterior spiracle specific expression we compared *crb43*.*2* sequence among several *Drosophila* species (S2B Fig). As the initial characterization of *crb43*.*2* indicated the requirement of two low affinity STAT binding sites [14], we tested a highly conserved 518 bp genomic fragment centred around these sites (S2B Fig). The 518 bp element was cloned into a *lacZ* reporter transformation plasmid containing a minimal promoter and *lacZ* expression was analyzed in transgenic flies. Like the original *crb43*.*2* enhancer, *crb*518 directs expression of the transgene specifically in the posterior spiracles (S2C Fig). Moreover, the activity of *crb*518 is also regulated by JAK/STAT signalling as both inactivation of the pathway or mutation of the STAT binding sites result in the downregulation of the enhancer’s activity (S2E and S2F Fig).

These results show that the *crb*518 enhancer constitutes a minimal element able to direct specific expression of *crumbs* to the posterior spiracle in a JAK/STAT dependent manner.

### A posterior spiracle specificity element can be separated from the STAT binding sites

**Fig 1:**
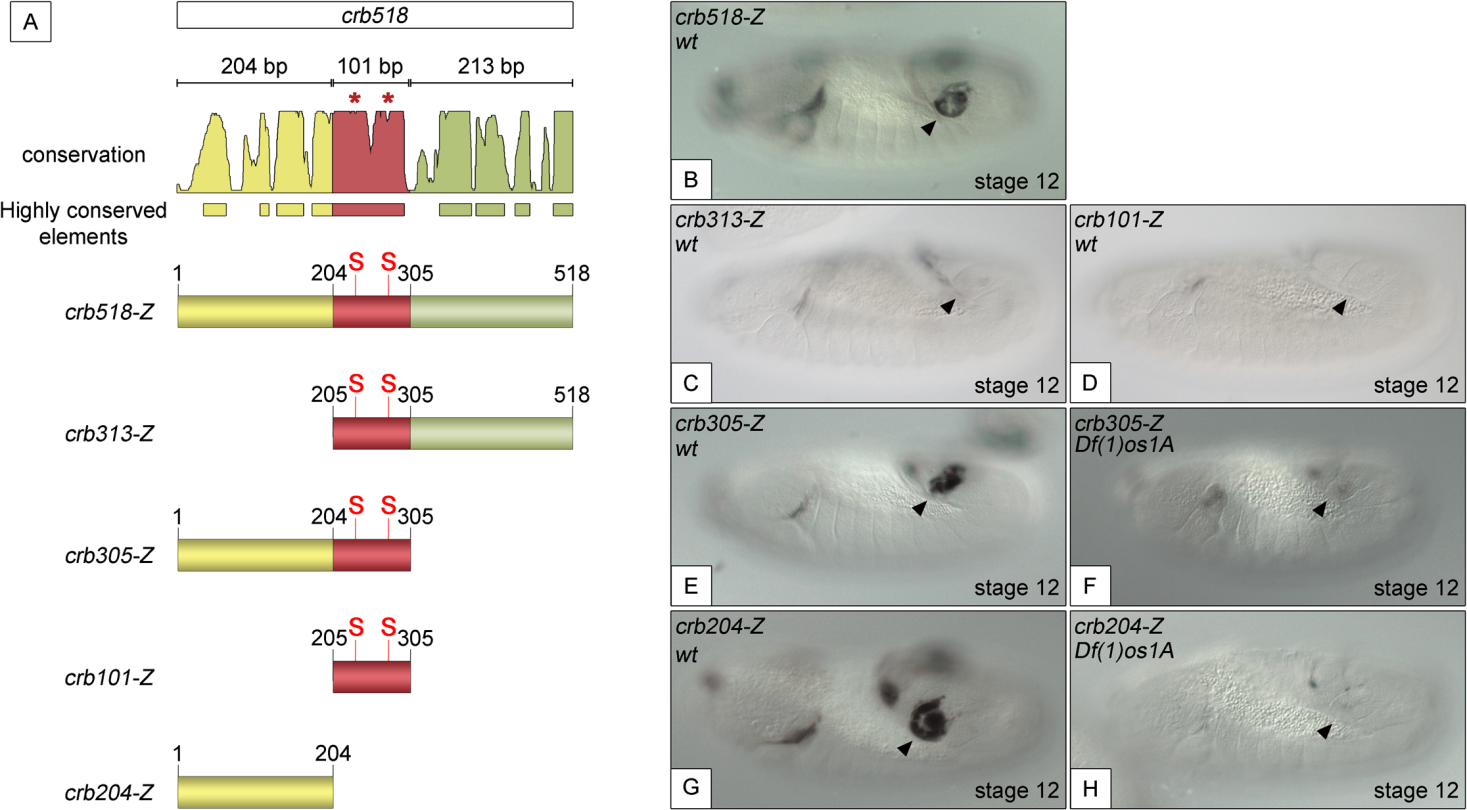
Component modules of the *crb518* posterior spiracle minimal enhancer. (A) Constructs designed to analyse the *crb518* CRM (S and asterisks indicate the position of STAT92E binding sites). (B) *crb518*-*lacZ* reporter expression. (C) The *crb313*-*lacZ* reporter with the 1–204 bp region deleted loses posterior spiracle expression. (D) The *crb101*-*lacZ* reporter containing the STAT92E binding sites is unable to direct expression to the posterior spiracles. (E) The *crb305-lacZ* reporter is able to direct expression to the posterior spiracle and its expression is downregulated in embryos mutant for the Unpaired JAK/STAT ligands (F). (G) The *crb204-lacZ* reporter lacking the STAT92E binding sites is able to direct expression in the posterior spiracles and still requires JAK/STAT function as in *unpaired* null mutants its activity in the posterior spiracles is abolished (H). Arrowheads: posterior spiracle primordium.

To understand the molecular mechanisms by which STAT activates *crumbs* in the posterior spiracles, we further dissected the *crb*518 minimal enhancer. Based on the position of the STAT binding sites, the *crb*518 enhancer can be subdivided into three regions (Fig 1A): a region encompassing the first 204 nucleotides (1–204, yellow), a 101 bp region containing the STAT binding sites (205–305 bp, red) and a region including the last 213 nucleotides (306–518 bp, green). Transgenes were designed with different combinations of these regions and their expression patterns characterized. When the 1–204 bp region is deleted (*crb313*), spiracle expression is lost (Fig 1C). This result suggests that the STAT binding sites although required, are not sufficient to direct expression to the posterior spiracles and that additional factors binding to the 1–204 fragment (yellow) are crucial. This conclusion is further supported by the inability of the *crb101* construct, which contains the 205–305 fragment (red) including both STAT sites, to direct expression in the spiracles (Fig 1D).

These observations are in accordance with our finding that the *crb305* enhancer, where the 306–518 fragment (green) is deleted, is able to direct expression to the posterior spiracle (Fig 1E). Moreover, the activity of *crb305* is still dependent on the JAK/STAT pathway as its expression is downregulated in the absence of all three *unpaired* ligands (Fig 1F). Therefore the *crb305* enhancer seemed to contain all the *cis*-regulatory elements required to direct expression to the posterior spiracle in a JAK/STAT dependent manner.

Finally, we tested the expression of the *crb204* construct, which contains the 1–204 fragment (yellow) but lacks the STAT binding sites. Surprisingly, the *crb204* is able to direct expression to the posterior spiracles (Fig 1G). Moreover, this expression is dependent on JAK/STAT pathway activity as in embryos mutant for the *unpaired* ligands the enhancer is downregulated (Fig 1H).

Taken together, these results show that *crb204* contains the *crb* posterior spiracle specificity element. Moreover, the expression of this construct suggests that although the activity of the JAK/STAT pathway is required to activate the *crb* spiracle expression, the STAT binding sites are dispensable. This seems to contradict our previous observations where mutation of the STAT sites resulted in the downregulation of the larger *crb43*.*2* and *crb518* enhancers.

### The spiracle specific *crb204* element does not contain cryptic STAT binding sites

The spiracle expression of the *crb204* enhancer was unexpected not only because it did not contain the STAT binding sites known to be required for the larger enhancers’ activity but also because despite the absence of STAT sites the enhancer is still regulated by the JAK/STAT pathway. These results prompted us to consider the possibility of cryptic STAT binding sites in *crb204* that could account for these observations. Therefore, we searched for putative additional STAT binding sites that differed in one or two nucleotides from the consensus TTCNNN(N)GAA sequence [14,15]. Using this relaxed criterion, we identified fifteen possible cryptic binding sites spread along *crb305* (S3A Fig, asterisks), twelve of them in *crb204*. To determine if STAT is able to bind *in vitro* to any of these putative sites, EMSAs were performed with activated STAT92E protein incubated with oligonucleotides containing the different cryptic sites (S3B Fig). As controls we used oligonucleotides containing the STAT consensus binding sites as well as mutated versions of these sites (Materials and Methods). In these conditions, STAT92E is only able to bind the previously identified consensus sites, while it fails to bind the mutated STAT sites or any of the putative cryptic sites (S3B Fig). Therefore, these experiments exclude the possibility of cryptic binding sites being responsible for the activity of *crb204*.

Taken together, these results suggest that the JAK/STAT pathway is required to specify expression in the posterior spiracles most likely by indirectly regulating factors binding to the 1–204 specificity element (yellow). This detailed analysis reveals that STAT does not function as a *crb* spiracle enhancer coactivator, but has a different function in the context of the *crb* enhancer.

### The binding of STAT92E to *crb*518 is required to counteract a repressor element

**Fig 2:**
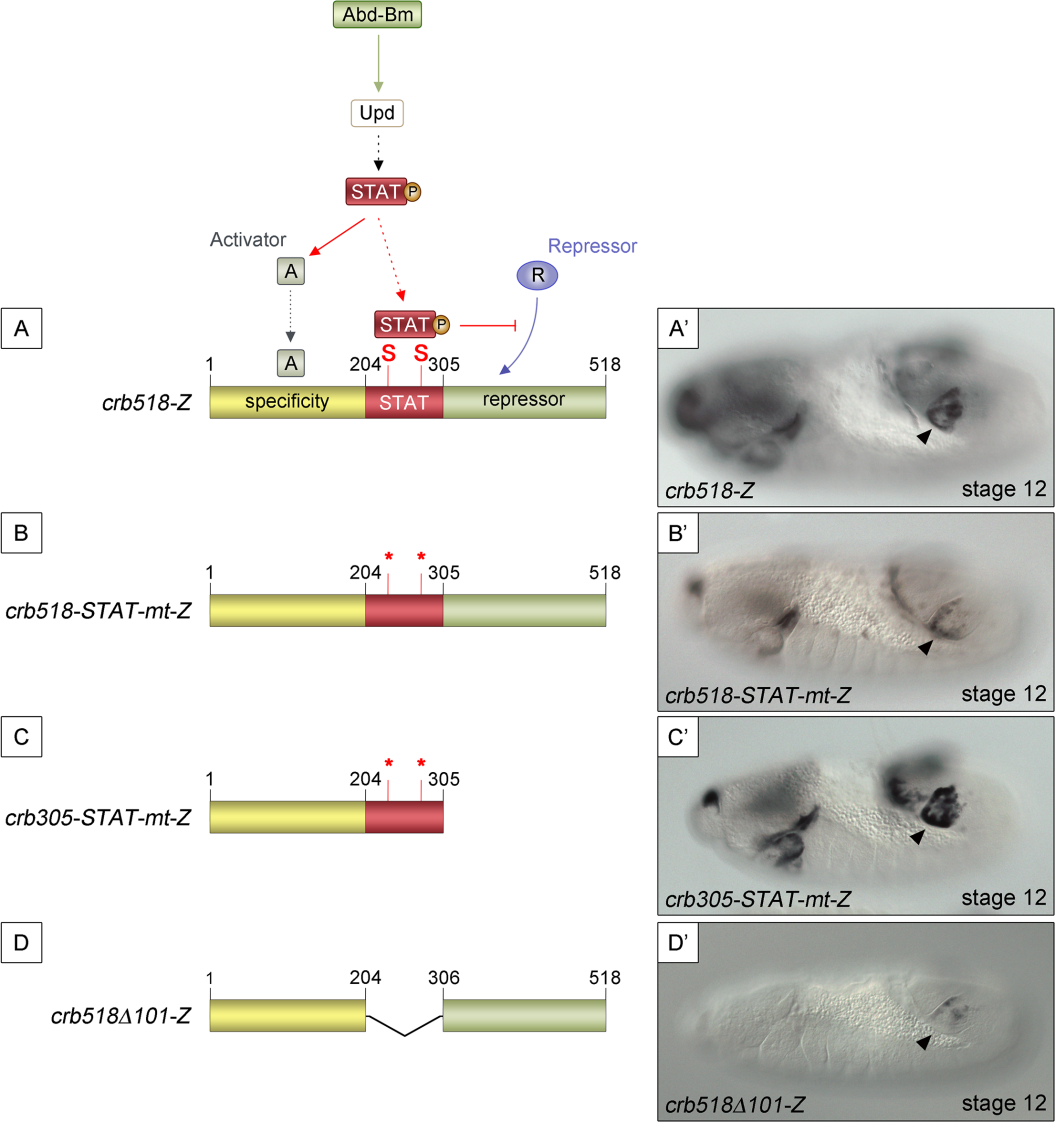
STAT92E binding sites are required to release *crb518* from repression. (A) Representation of the dual STAT92E requirement for *crb518* CRM expression in the posterior spiracles (A’). *crb518* requires direct STAT92E binding to counteract a repressor, as well as an indirect STAT92E function to induce an activator of the spiracle specificity module. (B) Mutation of the STAT binding sites (*crb518*-STAT-mt-Z) in the presence of the repressor module downregulates spiracle expression (B’). (C) Mutation of the STAT binding sites in the absence of the repressor module (*crb305*-STAT-mt-Z) does not affect spiracle expression (C’). (D) Fusion of the specificity and repressor modules in the absence of the STAT-binding module (*crb518* **Δ** *101*-Z) downregulates spiracle expression (D’). Arrowheads: posterior spiracle primordium.

Mutation of the STAT sites in both the original *crb43*.*2* enhancer [11,14] and in the *crb518* enhancer indicated that the STAT sites are required for full activation (S1E and S1F and S2C–S2F Figs). However, the spiracle activity of *crb204* excluded the possibility that the direct binding of STAT is necessary to direct expression to the posterior spiracles (Fig 1G). These contradictory results can be accommodated in a model where the specificity element is silenced by a nearby repressor element; the function of STAT being to prevent the repression, thereby, allowing spiracle specific activation (Fig 2A and 2A’). In this hypothesis the JAK/STAT pathway would play a dual role: first, it would be required indirectly to activate the specificity element by regulating activator factors; and second, it would be required directly to overcome the repression of the enhancer by counteracting a repressor element present in the CRM. Mutation of the STAT sites would prevent the binding of STAT to the CRM disabling this second counter-repression role resulting in the downregulation of expression controlled by the specificity element.

As our model predicts the existence of a repressor element, we set to identify its location by deletion of DNA in a *crb518* enhancer with mutated STAT sites (Fig 2B and 2C). The logic behind this experiment was that the deletion of the repressor element should allow the activity of the specificity element in the absence of STAT binding sites. We found that removal of the 306–518 fragment (*crb305*-STAT-mt) results in the expression of the enhancer in the posterior spiracles (Fig 2B’ and 2C’) confirming the existence of a repressor element in the 306–518 distal region (green region).

To further support our findings we generated an additional construct fusing the putative repressor module to the *crb* spiracle specificity module (Fig 2D). As expected from the presence of a repressor element, the expression of this transgene in the posterior spiracles is downregulated (Fig 2D’).

**Fig 3:**
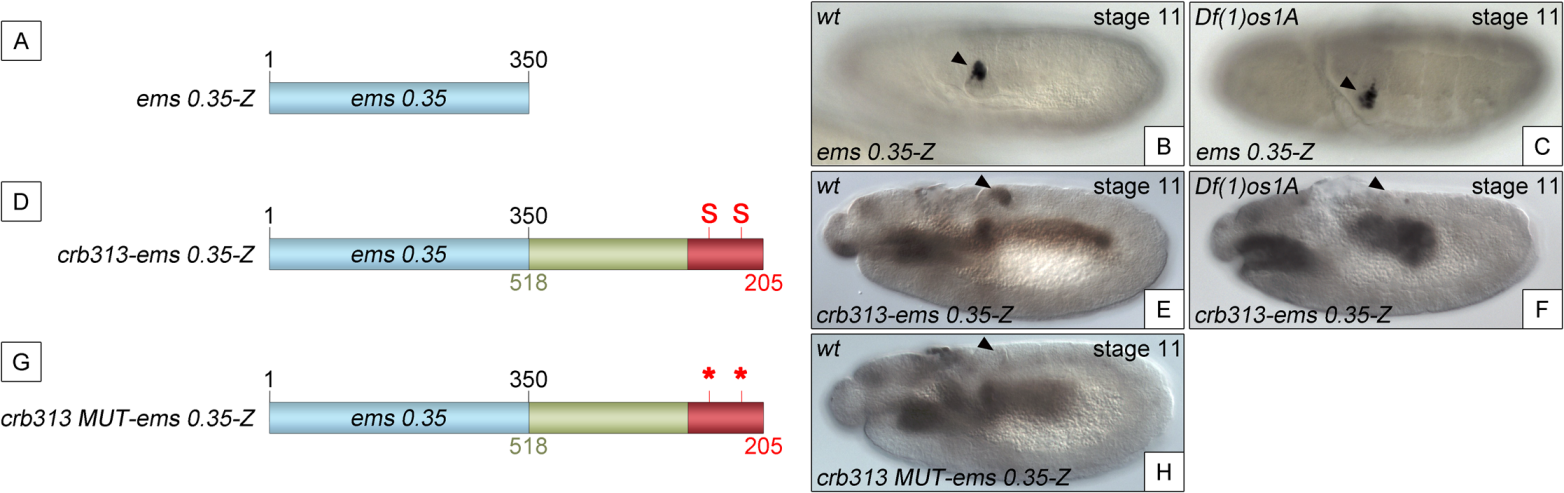
The repressor domain functions as an independent regulatory module. (A) The *ems0*.*35* enhancer (blue) drives expression to the posterior spiracles (B). (C) Expression of *ems0*.*35-Z* is maintained in *Df(1)os1A* mutant embryos lacking JAK/STAT pathway activity. (D) Fusion of the *crb* STAT-binding and repressor modules (red and green) to *ems0*.*35* (*crb313-ems0*.*35)* does not affect spiracle expression in wild type embryos (E), but abolishes spiracle expression in *Df(1)os1A* embryos (F). (G) When the STAT-binding sites are mutated in this construct (*crb313 MUT-ems0*.*35*) spiracle activity is lost (H). Note that the *crb313-ems0*.*35* fusion construct generates a novel gut pattern of expression that is STAT independent as it is unaffected in *Df(1)os1A* mutants or when the STAT sites are mutated. Arrowheads: posterior spiracle primordium.

To determine whether the STAT binding sites and the repressor element present in the 205–518 fragment are able to function as regulatory modules independent of the specific context of the *crb* spiracle specificity module, we fused the *crb313* fragment (red and green in Fig 1A), which does not drive expression in the posterior spiracles (Fig 1C), to *ems0*.*35* (Fig 3A and 3B), an unrelated posterior spiracle enhancer of the *empty spiracles* gene [16]. Although the *crb313-ems0*.*35* fusion construct generates a novel gut pattern of expression not present in any of the original constructs, *ems0*.*35* spiracle activity is still present when fused to the *crb313* element (Fig 3A and 3B and 3D and 3E). In contrast, fusion of the *crb313* element with the STAT sites mutated results in the loss of the posterior spiracle expression driven by *ems0*.*35* (Fig 3G and 3H). These results further support the presence of a repressor element in the 306–518 fragment that would be counteracted by the STAT sites, and reveal the modular nature of the *crb518* CRM. Moreover, while the *ems0*.*35* enhancer by itself is expressed independently of STAT function (Fig 3B and 3C), it becomes STAT dependent when fused to the *crb313* element (Fig 3E and 3F). Taken together, these data show that *crb518* is structured into three different regions, each with a specific function: an activator element driving spiracle specificity (1–204, yellow), a STAT-binding element that contains the conserved STAT binding sites (205–305, red) and a repressor element (306–518, green).

Analysis of the 306–518 DNA sequence did not provide any strong indication regarding the nature of the repressor protein. To fine map the repressor region, we constructed transgenes where we added increasing size fragments distal to *crb305*-*STAT-mt* (S4B Fig). In these constructs the position of the repressor element should be identifiable by its ability to inhibit the specificity spiracle element, which cannot be counteracted by STAT binding. Based on the sequence conservation between *D*. *melanogaster* and *D*. *virilis* (S4A Fig) the repressor containing region was divided into four elements: two highly conserved elements (CE), CE1 (345–385 bp) and CE2 (492–518 bp), intercalated by two non-conserved (NC) elements, NC1 (305–344 bp) and NC2 (386–491 bp). Addition of NC1 and CE1 has no effect on the activity of the enhancer (S4B–S4D Fig). However, when the NC2 element is included, the spiracle expression is downregulated (S4E Fig).

Taken together, these results map the position of the repressor element around the NC2 region distal to the STAT binding sites.

### The repressor element regulates the temporal activation of the *crumbs* spiracle enhancer

**Fig 4:**
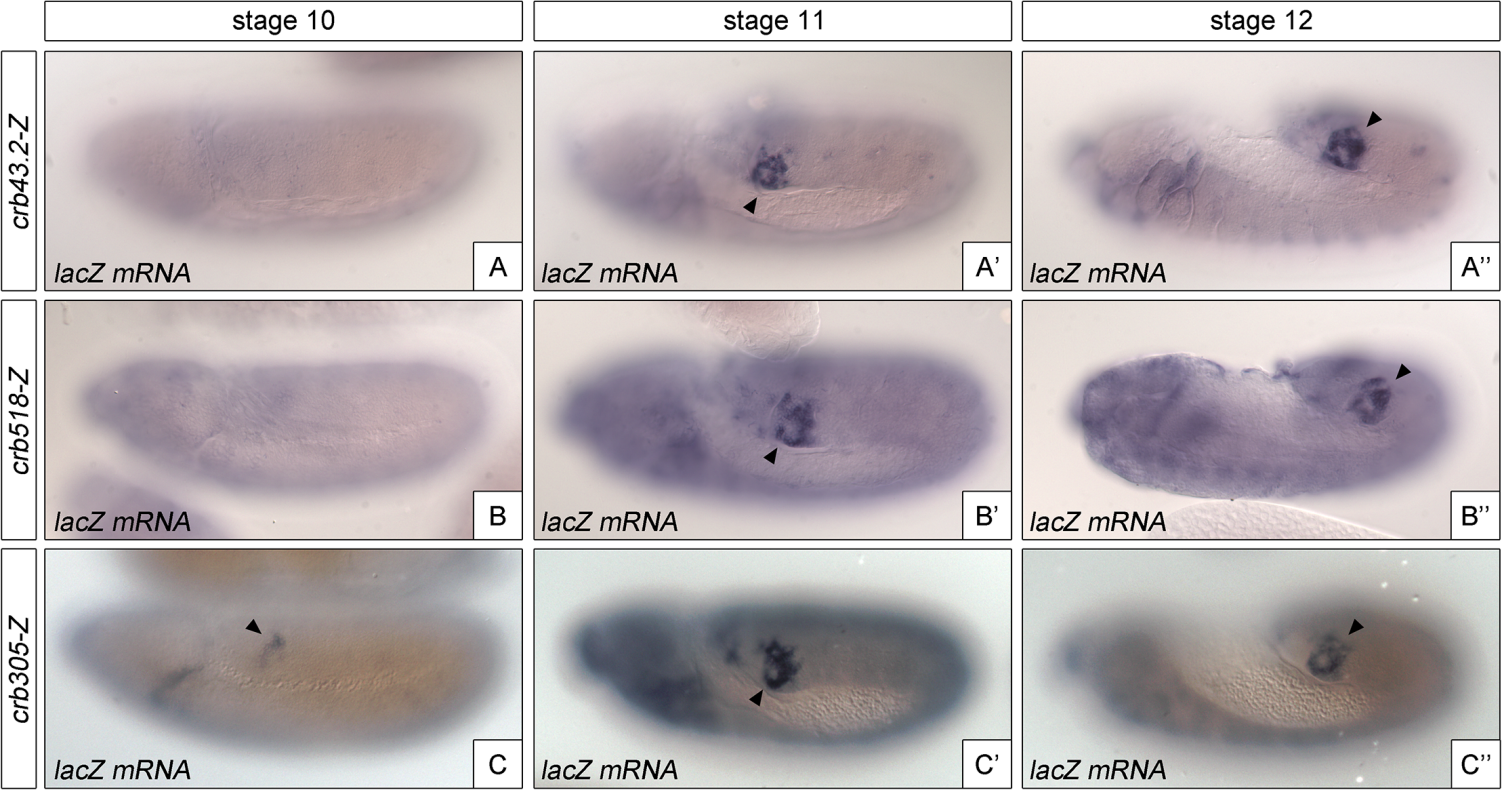
The repressor module regulates the temporal activation of *crb518*. Activation of the *crb* enhancer variants determined by *in situ* using *lacZ* RNA probes. While the spatial expression of the three reporters is similar, expression of *crb305* (C-C”) starts at an earlier developmental stage than *crb43*.*2* (A-A”) and *crb518* (B-B”), indicating that the repressor element regulates *crb*’s CRM temporal activation (compare A and B with C). Arrowheads: posterior spiracle primordium.

The identification of a repressor element in the *crb518* enhancer prompted us to determine its role in the regulation of *crb* in the posterior spiracle. The repressor domain does not seem to be involved in tissue-specific regulation as the specificity element (*crb204*, Fig 1G) is sufficient to direct spiracle activation. In fact, we were unable to observe any ectopic activation in constructs where the repressor element was deleted. Therefore, it seemed plausible that the repressor element could be required for temporal regulation of *crumbs* expression in the posterior spiracles. To test this possibility, we analyzed the onset of expression directed by the different *crb* CRM variants (Fig 4). While *crb43*.*2* and *crb518*, induce reporter transcription from stage 11 (Fig 4A'–4A”, 4B’–4B”), we observe that *crb305*, which lacks the repressor element, induces earlier transcription starting at stage 10 (Fig 4C–4C”).

These results demonstrate that the repressor element does not have a major function on tissue-specificity as expression is still detected in the posterior spiracles, but it does affect the temporal regulation of the enhancer with the onset of transcription occurring at earlier stages of development.

### Identification of trans-activating factors controlling *crb* spiracle specific activation

**Fig 5:**
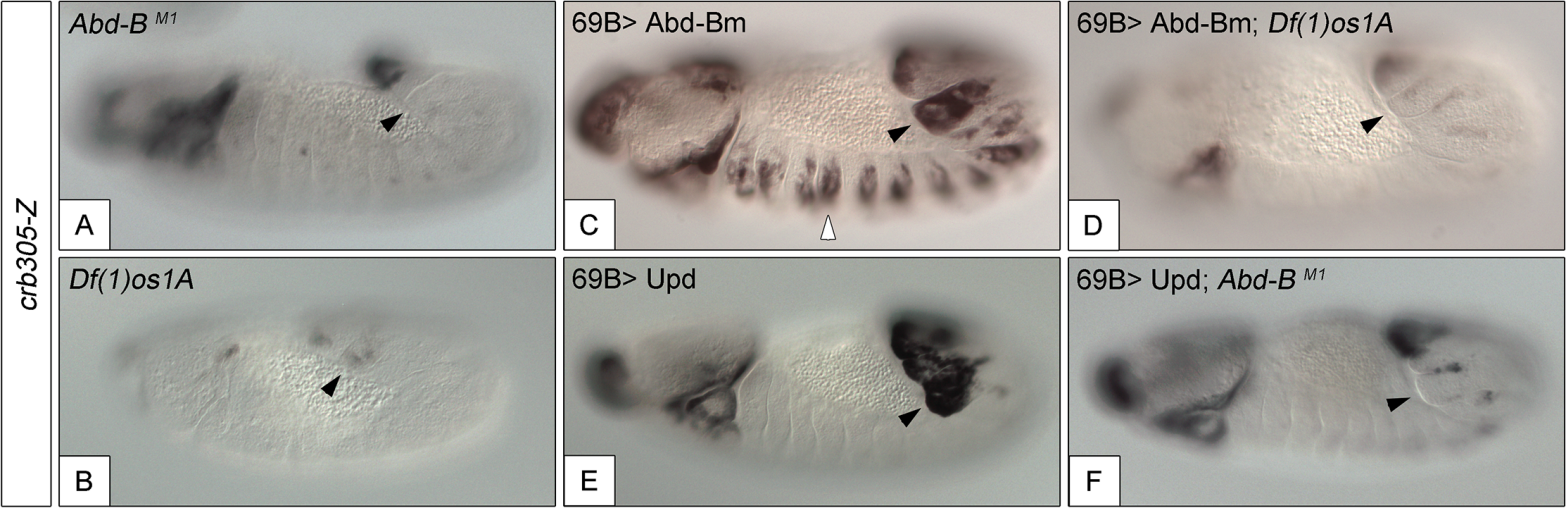
Activation of the *crb305* posterior spiracle enhancer requires a both Abd-Bm and STAT92E function. Expression of *crb305-Z* in *Abd-B* ^*M1*^ null homozygous mutant embryos (A), or *Df(1)os1A* embryos lacking all three Upd ligands (B). Ectopic expression of Abd-Bm in the ectoderm drives ectopic activation of *crb305* in all trunk segments in wild type embryos (C) but fails to do so in embryos lacking the Upd ligands (D). Ectopic expression of Upd, induces ectopic activation of *crb305* exclusively in the posterior segments (E). This activation is dependent on Abd-B function since ectopic expression of Upd in *Abd-B* ^*M1*^ mutant embryos fails to activate *crb305* (F). Arrowheads: posterior spiracle primordium.

**Fig 6:**
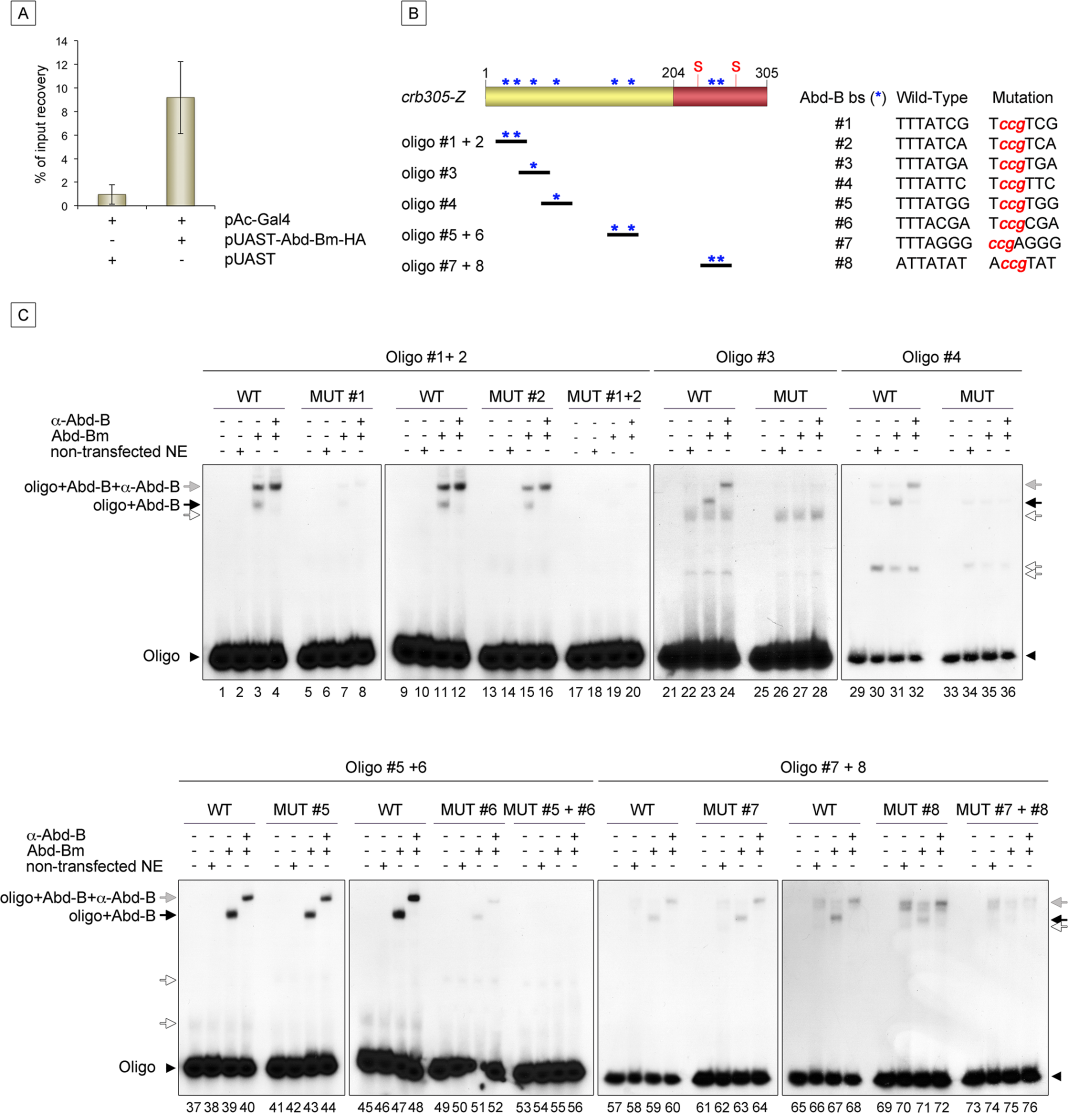
Abd-Bm binds multiple sites in *crb305* posterior spiracle enhancer. (A) Chromatin immunoprecipitation shows Abd-B binding to *crb* in S2 cells. Cells were transfected with pAC-Gal4 to activate the expression of UAS constructs, and either with pUAST-Abd-Bm-HA or empty pUAST as a control. ChIP was performed using an anti-HA antibody. (B) Localization of putative Abd-B binding sites in *crb305* (asterisks) and oligos used to test direct binding of Abd-B in EMSAs; (S) indicates the position of the consensus STAT92E binding sites. (C) EMSAs showing binding of Abd-B to wild type oligos (lanes 1–4, 9–12, 21–24, 29–32, 37–40, 45–48, 57–60, 65–68, black arrows) but not to oligos with the mutated sites (lanes 5–8, 13–20, 25–28, 33–36, 41–44, 49–56, 61–64, 69–76). In oligos containing two Abd-B sites, both have to be mutated to abolish Abd-B binding (lanes 5–20, 41–56, 61–76). Specificity of the observed band shifts was determined by the presence of a super-shift after incubation with anti-Abd-B antibody (grey arrows). White arrows point at unspecific bands. Protein extracts from non-transfected S2 cells were used as negative control. Arrowheads indicate the unbound radioactively labelled oligos.

Our finding that STAT is not involved in the direct activation of the *crb* spiracle enhancer, made us look into the posterior spiracle gene cascade for alternative activators. To this end, using the *69B-Gal4* line, we tested whether any of the Abd-B primary targets expressed throughout the epidermis could activate ectopic *crb305* expression. While ectopic expression of *spalt* (*sal*), *empty spiracles* (*ems*) or *cut* (*ct*) have no effect, ectopic expression of *unpaired* resulted in ectopic activation of the spiracle enhancer (Figs 5E and S5), consistent with the requirement of JAK/STAT for *crb* expression in the posterior spiracles. Interestingly, despite *69B-Gal4* expressing *unpaired* throughout the embryonic epidermis, ectopic activation of the enhancer is mostly confined to the posterior segments where Abd-B is also expressed. This result raised the possibility of Abd-B having a direct input on the activation of the *crb* specificity element. To test this possibility, we determined the ability of Abd-B to bind to the *crb* CRM in S2 cells using ChIP analysis. We obtained a 9-fold increase in input recovery when performing ChIP with Abd-B confirming that Abd-B is able to bind directly to the *crb* enhancer (Fig 6A).

Analysis of the *crb305* sequence shows the existence of eight putative Abd-B binding sites (Fig 6B, asterisks). To determine if Abd-B could bind them directly, EMSAs were performed with oligonucleotides covering regions containing the predicted sites (Fig 6B). As a control, we designed oligonucleotides containing point mutations abolishing Abd-B binding. Abd-B was expressed in S2 cells and cell extracts prepared and used in these assays. As seen in EMSA, all eight sites are bound by Abd-B with different affinities (Fig 6C). Moreover, binding to oligonucleotides containing more than one Abd-B site, is only abolished when both sites are mutant. A similar additive binding has been described for other direct Abd-B targets [16–18].

### JAK/STAT and Abd-B collaborate for *crb* spiracle enhancer activation

Our results suggest that Abd-B may be required both directly and indirectly for the activation of *crb* in the posterior spiracles. Directly, through Abd-B’s binding to the *crb* specificity element and indirectly through its activation of *upd* transcription and thus JAK/STAT signalling.

Similarly, STAT is also required directly and indirectly for *crb*’s spiracle expression: directly, to block the repressor’s element activity; and indirectly, because JAK/STAT signalling is also required for the activation of the *crb* specificity element (Fig 1H).

To study if Abd-B can induce the activation of the specificity domain in the absence of JAK/STAT signalling and vice versa, we analyzed the effect of ectopically expressing one in embryos mutant for the other. We performed these experiments using the *crb305* enhancer that lacks the repressor domain, avoiding the interference of the repressor element. The ectopic *crb305* expression in segments A8-A9 caused by ectopic *upd* is not present in *Abd-B* mutants (compare Fig 5E and 5F) showing that Abd-B provides an additional input in the activation of the *crb305* enhancer. Similarly, ectopic Abd-B expression in *Df(1)os1A* embryos lacking all three *upd* ligands is not capable of activating the *crb305* enhancer (compare Fig 5C and 5D). Therefore, these results indicate that both STAT and Abd-B inputs are necessary in parallel for spiracle specificity rather than all the Abd-B activity being mediated by its activation of *upd* and JAK/STAT signalling.

### JAK/STAT activates expression of Abd-B via a feedback loop

The above data show that both STAT and Abd-B are required for *crb* specificity element expression. As the function of STAT on the specificity element is indirect, it is likely to be mediated through the regulation of another transcription factor expressed in the posterior spiracles. This means that a still unidentified STAT target mediates STAT’s indirect function on the *crb* spiracle enhancer. Alternatively, Abd-B itself could be regulated by STAT via a feedback mechanism.

**Fig 7:**
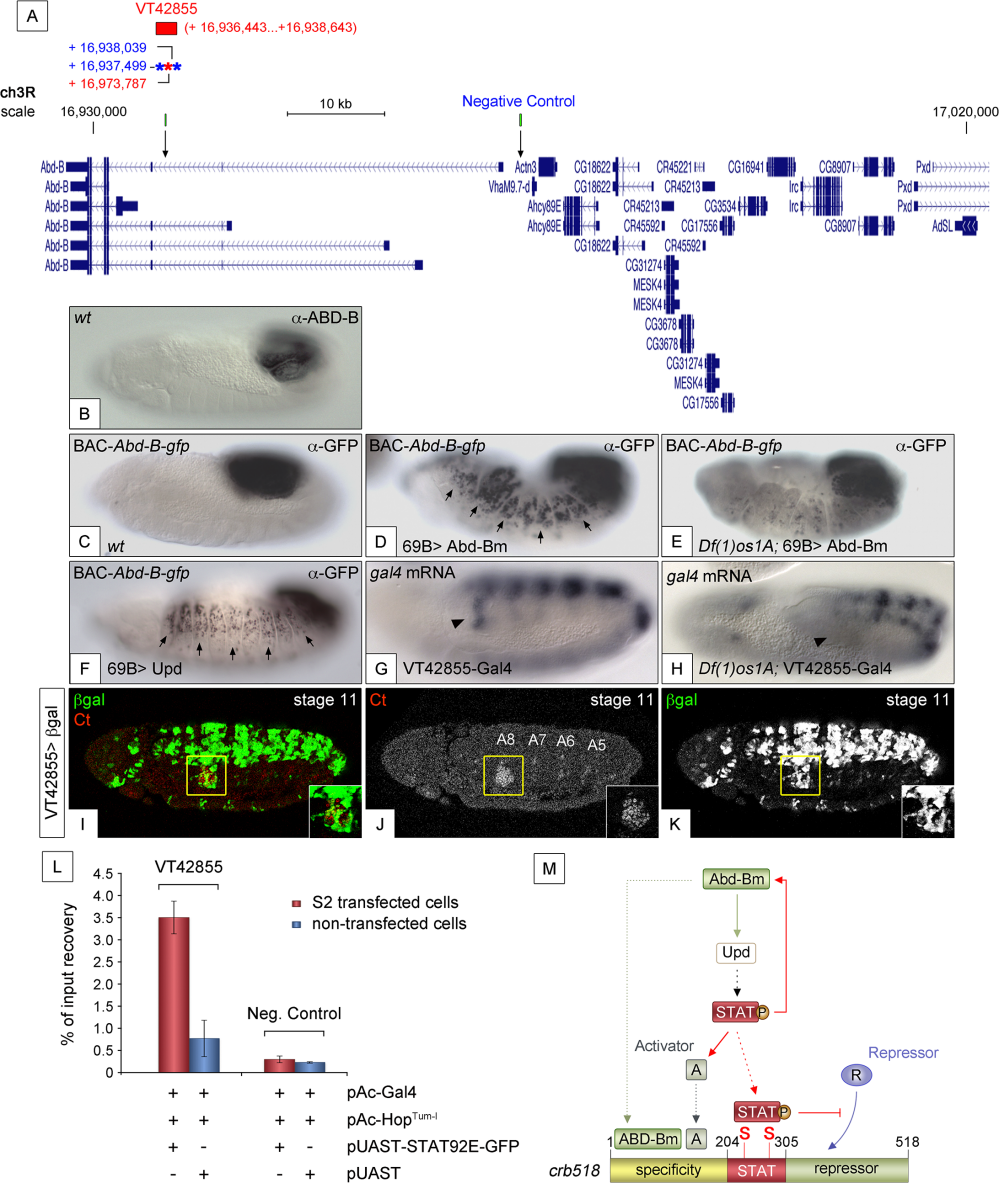
JAK/STAT activates expression of Abd-B through a feedback loop. (A) Genomic region contained in [CH321-91P18] BAC-*Abd-B-gfp* and location of the *VT42855* enhancer sequence. Asterisks represent 3N (red) and 4N (blue) STAT sites present in the *VT42855*, green boxes and arrows in the scale line mark the position of the control and experimental oligos used in the ChIP experiment below. (B) Endogenous Abd-B protein expression. (C) GFP-tagged Abd-B expression from the BAC-*Abd-B-gfp* is indistinguishable in the A8 and A9 abdominal segments from the endogenous protein. (D) Ectopic Abd-Bm expression in the embryonic ectoderm using *69B-Gal4* induces ectopic BAC-*Abd-B-gfp* activation anterior to A8. (E) In *Df(1)os1A* embryos lacking JAK/STAT ligands ectopic Abd-Bm expression cannot activate high levels of BAC-*Abd-B-gfp* anterior to A8. (F) Ectopic Upd expression using *69B-Gal4* induces ectopic BAC-Abd-B-gfp activation. Note in D and F that the expression observed in A8 and A9 is stronger than the ectopic expression in anterior segments. This is likely due to the BAC element containing several A8-A9 enhancers not all of which will respond to the feedback and become activated ectopically. (G) Posterior spiracle *gal4* RNA expression (arrowhead) driven by the 2.2kb Abd-B genomic fragment in VT42855-Gal4 line. (H) Posterior spiracle expression in *VT42855-Gal4* disappears in *Df(1)os1A* embryos lacking JAK/STAT ligands. Note that central nervous expression is unaffected. (I-K) *VT42855-Gal4/+*, *UAS-lacZ/+* embryos double stained with anti-**β**Gal (green in I, white in K) and anti-Ct (red in I white in J) to prove the posterior spiracle expression (inset) of *VT42855*. (L) STAT92E-GFP Chromatin immunoprecipitation in S2 cells shows STAT binding to the *VT42855* region of the *Abd-B cis* regulatory element compared with the negative control region devoid of STAT sites indicated in A (green boxes). Cells were transfected with pAC-Gal4 to activate the expression of UAS constructs, pAC-Hop^Tum-l^ expressing an activated JAK kinase to induce STAT92E activation by phosphorylation and either with pUAST-STAT92E-GFP or empty pUAST as a control. ChIP of STAT92E was performed using an anti-GFP antibody. The position of oligos used to amplify the *VT42855* region is indicated in (A) by an arrow and their sequences indicated in Materials and Methods. (M) The *crb518* posterior spiracle enhancer is composed of a specificity module sufficient to drive posterior spiracles expression, a STAT binding module and a repressor module. Early Abd-B expression activates the JAK/STAT Unpaired ligand and other primary posterior spiracle targets, but fails to activate *crb518* CRM as a result of the repressor module activity. The JAK/STAT pathway plays a key role in the activation of *crb* by, first, reinforcing Abd-B expression in A8 through a feedback loop mechanism; second, inducing additional activator factors, which together with ABD-B act over the specificity module and finally by direct binding of STAT92E to *crb518* to counteract the repressor element. Arrowheads in G and H indicate the position of the posterior spiracle primordium; arrows in D and F indicate some of the segments that exhibit ectopic activation of the BAC-*Abd-B-gfp*.

If a feedback loop mechanism existed in the posterior spiracles, overexpressing Abd-B should result in the activation of endogenous *Abd-B*. To determine the existence of such feedback loop, the *69B-*Gal4 line was used to ectopically activate *UAS-Abd-Bm* in the ectoderm of embryos containing the *BAC-Abd-B-GFP* (Fig 7A). In this BAC element *GFP*-tagged *Abd-B* is regulated by the endogenous upstream *Abd-B cis*-regulatory sequences that are sufficient to drive normal expression in the A8 and A9 segments (Figs 7B and 7C and S6A–S6A”). This set-up allows discriminating between the ectopically expressed Abd-Bm protein and any feedback induced Abd-B-GFP protein from the *BAC* element. Ectopic *UAS-Abd-Bm* induction results in ectopic expression of Abd-B-GFP, indicating that a feedback loop mechanism maintaining Abd-B expression does exist (Fig 7D). We next tested whether this feedback loop could be mediated by the activation of the JAK/STAT pathway. To determine this, we repeated the experiment in a *Df(1)os1A* mutant background and found that most feedback-induced ectopic expression disappeared (Fig 7E) while feedback-independent expression in A8-A9 is maintained. We also ectopically expressed *upd* throughout the ectoderm using the *69B-Gal4* driver line and analyzed Abd-B-GFP expression. As with ectopic expression of Abd-Bm, Upd also results in the ectopic activation of Abd-B-GFP expression (Fig 7F). As these results suggest the existence in Abd-B of an enhancer regulated by JAK/STAT signalling, we analyzed the Stark Lab genome collection of enhancers made from the *Abd-B* locus [19]. We found that a 2.2 kb fragment (VT42855) drives expression in the posterior spiracles and contains a high density of STAT sites (Fig 7A and 7G and 7I–7K). Chromatin immunoprecipitation with STAT-GFP shows an enrichment of input recovery of this fragment suggesting that STAT is able to bind directly to the *Abd-B* cis regulatory region (Fig 7L). Moreover, *VT42855* expression in the posterior spiracles is abolished in *Df(1)os1A* mutants (Fig 7G and 7H) confirming the existence of a JAK/STAT regulated enhancer in the Abd-B locus. Taken together, these results indicate the existence of a feedback loop mechanism used to maintain spiracle expression of Abd-B via activation of the JAK/STAT pathway.

## Discussion

Using the posterior spiracles as a model, we have analysed how an organogenetic gene network develops and how a particular gene of this network integrates the changing genetic information.

### Dynamics of the global genetic regulatory interactions during organogenesis: transformation of a genetic cascade into a gene network

Two main regulatory structures have been described during organogenesis: genetic cascades and genetic networks. Genetic cascades are characterized by the hierarchical interactions between its components, with one gene setting the stage and the others responding to it. In this system, the type of organ to develop is chosen by the upstream *selector* gene, which controls the different cell behaviours via intermediate transcription factors [8,20–22].

Gene networks differ by the absence of a single *master* gene. In a gene network, like that controlling eye development, not only several genes can trigger organogenesis but also all of these genes are necessary for organogenesis. In the absence of anyone of them, organogenesis cannot proceed, even if forced expression of the other master regulators is induced. [23–25]. This behaviour suggests that none of the regulators is exclusively upstream of the others and that activation of any of the master genes results in the activation of the other master genes through cross-regulatory loops [9]. Cross-regulatory feedback loops confer robustness to a regulatory system and their absence in early Hox induced organogenesis could be due to the fast development of the *Drosophila* embryo that precludes Hox genes to become integrated by feedback loops into the cascades they activate.

The formation of the posterior spiracle is a typical example of organogenesis induced by a Hox gene cascade [11]. Here, Abd-B activates the primary targets that, in turn, activate secondary targets like Cad96C, Cad88C, Gef64C and Cad74A. The hierarchical organization of these targets was demonstrated by the observation that in *Abd-B* mutants the simultaneous activation of the four Abd-B primary targets can activate the expression of *Cad96C*, *Cad88C*, *Gef64C* and *Cad74A* [11]. Despite of this, here we have found evidence that during embryonic development, the Abd-B spiracle cascade is already transforming into a gene network. We have observed that Abd-B activates the JAK/STAT signalling pathway in the posterior spiracles through *upd* and that the JAK/STAT pathway feedbacks to enhance Abd-B expression. Two observations show that this is important to maintain robust *crb* expression: first, JAK/STAT pathway signalling only weakly activates the expression of the *crb* posterior spiracle enhancer in segments that do not express Abd-B; and second, JAK/STAT pathway activation is insufficient to activate the *crb* enhancer in the A8 segment of *Abd-B* mutant embryos (Fig 5). An indirect feedback loop affecting the Ubx Hox gene has been reported [26]. In the visceral mesoderm Ubx activates in PS7 the signalling molecule dpp. Dpp signals to the neighbouring PS8 cells to activate with Abd-A wingless expression. In turn, Wg signals from PS8 back to PS7 where it is required for the maintenance of Ubx expression. This case and the JAK/STAT—Abd-B interaction we describe here may represent the earliest examples of indirect autoregulatory loops transforming a Hox-cascade into a Hox-network. It would be interesting to analyze other organogenetic cascades to find out if the establishment of feedback loops is a common theme during development and at what stage they become active.

### Integrating the global genetic regulatory information into single target genes: the posterior spiracle regulation of *crb*

During organogenesis the complex patterning information set by the transcription factors and signalling molecules upstream in the cascade has to be integrated to activate the realizator molecules modulating the organogenetic cell behaviours [27]. This is mediated through the *cis* regulatory modules of each realizator gene. In this study, we have focused on how the posterior spiracle gene cascade activates the *crb* polarity gene through a CRM that responds to JAK/STAT signalling. The observation that the spiracle enhancer requires the activity of *upd*, an Abd-B primary target, suggested that Abd-B activates *crb* indirectly, by setting STAT activity [11]. Our dissection of the *crb-spiracle* CRM has revealed that a spiracle specificity element can be separated from the STAT binding sites and that the “minimal” CRM is modular in nature. The *crb*-spiracle CRM studied here is composed by, at least, three independently acting elements: an activator element where the spiracle specificity resides; a STAT binding element that is only required to counteract a neighbouring repressor element; and a repressor element that interferes with the activity of the specificity module.

We have found that the spiracle specificity element is bound by Abd-B suggesting that *crb* is a direct target of Abd-B. However, *crb* is different from other Abd-B primary targets, since the *crb* specificity element also requires for its expression STAT activation, itself a target of Abd-B. Because of its mixed character as a direct and an indirect target, we term *crb* a delayed-primary target: primary to reflect its direct regulation by Abd-B, and delayed because it can only respond to the presence of Abd-B after other Abd-B primary targets are active. The requirement of both Abd-B and JAK/STAT signalling for *crb* activation explains why the *crb*-spiracle enhancer is only active in A8 and not in more anterior segments where JAK/STAT signalling is also active, as well as why *crb*-spiracle enhancer expression in A8 is restricted dorsally despite Abd-B being expressed throughout this segment.

The repressor element we have identified is “transplantable” and can function autonomously on an independent posterior spiracle enhancer. In fact, we have shown that the *ems* spiracle enhancer, that is STAT independent (Fig 3C), can be engineered to become STAT dependent by the inclusion of the STAT binding and repressor modules found in *crb* (Fig 3E and 3F). The observation that the specificity element in *crb* is sufficient to drive the activity of the enhancer, begs the question of why is there any need for the presence of further modules in the enhancer. As the specificity element cannot function until the repressor activity is counteracted by Abd-B induction of JAK/STAT signalling in the spiracle, the repressor module provides the CRM with a subtle system to delay the *crb* spiracle expression during development.

The integration of the results in this work uncovers the existence of complex Abd-B and JAK/STAT dynamic interactions that are described by the model shown in Fig 7M. Early Abd-B binding to the *crb* specificity module cannot activate transcription due to the presence of the neighbouring repressor module. This repression is relieved when Abd-B activation of *upd* in the posterior spiracles activates STAT. Direct binding of STAT to the *crb* CRM relieves the repression. We hypothesize that STAT induces the function of another activator protein (A) that also binds to the specificity module (Fig 7M). The existence of such activator driven downstream of STAT is necessary to explain why ubiquitous ectodermal Abd-B expression with the *69B-Gal4* line is unable to activate the *crb305* enhancer in *upd* mutant embryos despite the absence of the repressor module (Fig 5D). Besides these functions, our data show that STAT reinforces *Abd-B* expression in the spiracles through a feed back loop. The model in Fig 7M can explain why the inactivation of JAK/STAT signalling in *Df(1)os1A* embryos has a stronger effect on *crb518* enhancer expression than the deletion of the STAT binding sites (compare S2E and S2F Fig and similar observations using *crb43*.*2* in Lovegrove et al. [11]). Mutation of the two STAT binding sites decreases *crb* expression because the repressor module is free to interfere with the specificity module. Mutation of *Df(1)os1A* has a larger influence on expression because it affects *crb* regulation at three levels: First, because, as in the previous case, the repressor module is free to interfere with the specificity module in the absence of active STAT; second, because the proposed Activator downstream of JAK/STAT will not be activated; and third, because the STAT feedback loop over *Abd-B* will not be activated. Although we still do not know all the players involved in the regulation of the *crb* posterior spiracle enhancer, this case provides an example of how gene networks and CRMs dynamically interact during development.

The complex CRM controlling *crb* spatial and temporal regulation in the posterior spiracles provides a plastic regulatory platform that could be subtly modulated during evolution. Earlier *crb* activation could be obtained by loss of the repressor module and lower or delayed expression by removal of Abd-B or STAT binding sites. Such quantitative and temporal regulation changes in either direction could be rapidly established by selection of minor mutations in these elements.

### Counter-repression: an alternative way for STAT to control cell specific transcriptional regulation

STAT proteins in vertebrates and invertebrates are considered to be a family of latent transcription factors activated by phosphorylation. After their activation, the cytoplasmic proteins dimerize and accumulate in the nucleus where they bind DNA through TTC(3-4N)GAA sites to activate transcription [28,29]. STAT specific transcriptional activation has been suggested to require either cooperative interaction with other enhancer binding proteins, or interaction with co-activator proteins that stabilize or recruit RNA polymerase II complexes or destabilize chromatin [30,31]. In this view, transcription of specific downstream targets is due to the direct interaction of STAT with other activators expressed in the same cells. Initially, we expected the STAT dependent up-regulation of *crb* in the spiracles to be controlled using such a mechanism, however, we have found this not to be the case. Our experiments show that direct STAT binding to the posterior spiracle’s specificity element is not required for transcription. This result suggests that STAT does not function as a classical transcription factor stabilizing RNA polymerase II in the posterior spiracles, but rather acts as a counter-repressor protein. STAT function as a counter-repressor is to prevent that proteins binding to the repressor element interfere with the binding or function of transcriptional activators in the specificity module. Thus, we propose that besides its function as a transcriptional activator, STAT can control gene specific expression through a novel function: counter-repression. Although future work should identify the STAT92E domain controlling counter-repression, we can predict that if the counter-repression domain were different from the transactivation domain, deletion of the transactivation element would still result in a STAT92E protein capable of controlling transcription through counter-repression. The existence of an independent domain with such new function could explain published results showing that STAT proteins with a deleted transactivation domain are still active proteins and do not act as dominant negative transcription factors [32]. Counter-repression is not related to the non-canonical JAK/STAT control of heterochromatin stability described by Li and collaborators as this does not require direct STAT binding to its consensus DNA sites [33–35], while we show that STAT counter-repression requires the presence of its binding sites.

In summary, we have shown that the Hox protein Abd-B initiates a simple embryonic posterior spiracle cascade that soon evolves into a gene network. In this network, Abd-B and the JAK/STAT signalling pathway (which is activated downstream of Abd-B in the spiracles) interact to precisely control the temporal activation of the *crb* realizator gene. Importantly, our study uncovered a novel way for STAT proteins to control cell specific gene expression: permissive counter-repression.

## Materials and Methods

### Fly strains

The following Gal4 driver and UAS lines were used: *69B-Gal4* to drive expression in the whole ectoderm, *UAS-Abd-Bm*, *UAS-upd*, *UAS-ct*, *UAS-sal*, *UAS-ems*, *UAS-grn*. We used the *Abd-B* ^*M1*^ null allele, the *Df(1)os1A* [deleting all three Upd ligands, [36]]. As reporter spiracle lines we used the *crb43*.*2*.*1-lacZ* [11] and *ems0*.*35-lacZ* [16], while the *10xSTAT-dGFP* reporter was used to detect global JAK/STAT activation [37]. The *VT42855-Gal4* line was obtained from the Stark lab fly transcriptional enhancer collection http://enhancers.starklab.org/. The [CH321-91P18] *BAC-Abd-B-GFP* stock is a gift from Rebecca Spokony, modENCODE consortium. To test if the BAC can rescue Abd-B function, we combined *BAC-Abd-B-GFP* with a *Ubx* ^*MX12*^, *abd-A* ^*M1*^, *Abd-B* ^*M8*^ triple mutant [38] (S6 Fig).

### Immunohistochemistry and RNA *in situ* hybridization

The embryos were grown at 25°C except the 69B-Gal4 UAS overexpression experiments that were grown at 29°C to increase Gal4 activity. The following primary antibodies were used: mouse anti-AbdB 1A2E, anti-Crb and anti-Ct 2B10 (Developmental Studies Hybridoma Bank); rabbit anti-GFP (Invitrogen) and mouse anti-**β**Gal (Promega). Secondary antibodies were conjugated to Alexa Fluor 488, 555 (Invitrogen). Confocal images were obtained with a Leica SP2-AOBS microscope and processed using ImageJ and Adobe Photoshop CS5. We used antisense RNA probes for *upd*, *Gal4* and *lacZ in situ*.

### Constructs

The design of the constructs was based on the sequence conservation analysis of *crb43*.*2* by comparison of the twelve *Drosophila* species and *A*. *gambiae*, *A*. *mellifera* and *T*. *casteneum* genomes using the UCSC Genome Bioinformatics (http://genome.ucsc.edu/). All *crb* fragments were subcloned into the *pCasper-hs43-lacZ* transformation plasmid. To this end, *pCasper-hs43-lacZ* was digested first with EcoRI, the 5’overhang filled with Klenow and finally digested with EclXI. Unless stated otherwise, all PCR reactions were performed using as DNA template the 43.2-pBluescript-SK plasmid containing the *crb43*.*2* genomic region [11]. The primers used to generate the different constructs are listed in S1 Table.

To generate the *crb518-Z* construct, the 43.2-pBluescript-SK plasmid was digested with DraII, the resulting 5’overhangs filled with Klenow and subsequently digested with EclXI. A 518 bp fragment was isolated, purified and subcloned into *pCasper-hs43-lacZ*.

To construct *crb518-STAT-mt-Z*, mutation of the STAT binding sites was carried out in a two-step process. The first STAT site was mutated by site-directed mutagenesis as described in [39] using the *stat4n1-mt* primer. The resulting plasmid with the mutated first STAT binding site was then used as a template to perform the mutation of the second STAT site by PCR mutagenesis. A first PCR reaction was performed using the primer pair *crb-stat2-mt* and *rev-crb*. The product was purified and used as a reverse primer in a second PCR reaction using *fw-crb* as forward primer. The resulting PCR product containing both mutated STAT binding sites was digested with EclXI and subcloned into *pCasper-hs43-lacZ*.

The *crb305-Z*, *crb313-Z*, *crb101-Z*, *crb204-Z*, *crb305-STAT-mt-Z*, *crb* **Δ** *25-Z*, *crb* **Δ** *133-Z* and *crb* **Δ** *174-Z* constructs were generated as follows: the region of interest was amplified by PCR using either 43.2-pBluescript-SK (for *crb305-Z*, *crb313-Z*, *crb101-Z* and *crb204-Z*) or *crb518-STAT-mt* (for *crb305-STAT-mt-Z*, *crb* **Δ** *25-Z*, *crb* **Δ** *133-Z* and *crb* **Δ** *17*4-*Z*) as DNA template; the PCR products were digested wit EclXI and subcloned into *pCasper-hs43-lacZ*. The primer pairs used are as follow: *fw-crb* and *rev-stat* (*crb305-Z and crb305-STAT-mt*), *fw-stat* and *rev-crb* (*crb313-Z*), *fw-stat* and *rev-stat* (*crb101-Z*), *fw-crb* and *rev-crb204* (*crb204-Z*), *fw-crb* and *crb* **Δ** *25-rev* (*crb* **Δ** *25-Z*), *fw-crb* and *crb* **Δ** *133-rev* (*crb* **Δ** *133-Z*) *and fw-crb* and *crb* **Δ** *174-rev* (*crb* **Δ** *174-Z*).

To generate *crb518* **Δ** *101-Z*, a first PCR was performed using the primer pair *fw-* **Δ** *101* and *rev-crb*. The resulting product was purified and used as a primer in a second PCR with the *fw-crb* primer. The resulting PCR product was digested with EclXI and subcloned into *pCasper-hs43-lacZ*.

To generate the *crb313-ems0*.*35-Z* constructs PCR reactions were performed using the reverse primer *rev-crb* with *fw-crb205-BHI*. The PCR products were purified and digested with EclXI and cloned into *pCasper-ems 0*.*35* [16] digested with EclXI and BamHI.

All constructs were randomly inserted using P-element transformation. We analysed between three and ten independent lines to discard insertion-site position-effects. Injection was performed by the *Drosophila* Consolider-Ingenio (CBM-SO, Madrid) platform or by BestGene.

### Chromatin Immunoprecipitation (ChIP) assays

This protocol, based on [40], was done as previously described [14,16]. ChIP was performed using transiently transfected S2 cells. 1 x 10^7^ cells were seeded in 10 cm cell culture dishes one day before transfection. For Abd-B ChIP, cells were transfected with either (1) 5 **μ**g pUAST-Abd-Bm-HA and 5 **μ**g pAC-GAL4 or (2) 5 **μ**g of empty pUAST and 5 **μ**g pAC-GAL4 plasmids. For STAT92E-GFP ChIP, cells were transfected with either (1) 3.5 **μ**g pUAST-STAT92E-GFP, 3.5 **μ**g pAC-Hop^Tum-l^ and 3.5 **μ**g pAC-GAL4 plasmids or (2) 3.5 **μ**g empty pUAST, 3.5 **μ**g pAC-Hop^Tum-l^ and 3.5 **μ**g pAC-GAL4 plasmids. 1/10 of cells were collected to monitor the protein expression by Western blot. Remaining cells were cross-linked, lysed and sheared to 350–1000 bp. Immunoprecipitations were performed using 6 **μ**l of anti-HA antibody (Abcam) or 50 **μ**l of anti-GFP conjugated magnetic beads (MBL) per 100 **μ**g sheared chromatin. qRT-PCR was performed with primers crbQPCR1for (5’-TTCATTCATTTCCATGAACACA-3’) and crbQPCR1rev (5’-ATTCGTCGGTTTTCCTTGTC-3’) amplifying inside the *crb43*.*2* enhancer sequence; AbdB Bac1for (5’-TTGGACAAATTCACATGCAA-3’) and AbdB Bac1rev (5’-GGCCAATGAACTTCCCTCTA-3’) amplifying inside the *VT42855* enhancer sequence; and, as a control, with AbdB BacC-Afor (5’-TGAACTTAAATGCCGAATCAA-3’) and AbdB BacC-Arev (5’-CACAAGAAGTGCGTGACTGA-3’) amplifying in a sequence lacking STAT binding sites. The data are represented as recovered percentage from the input in HA-Abd-B-transfected cells or GFP-transfected cells against pUASt-empty-transfected cells.

### Electrophoretic mobility shift assays (EMSA)

The complementary oligonucleotides (Sigma Aldrich) used to generate the radiolabelled probes in EMSAs to determine STAT92E and Abd-B binding are listed in S2 and S3 Tables respectively. Radioactively labelled probes were generated by annealing and subsequent end filling with [**α**-^32^P]dCTP. The conditions used were similar to those described previously [16]. Briefly, double-stranded, end-labelled DNA (50,000 cpm/binding reaction; 10 nM) was incubated for 30 min at 4°C with 2 **μ**l of cell extract lysate expressing each tested protein or 2 **μ**l of the cell extract control and 50 mM NaCl, 5 mM EDTA, 0,5 mM DTT, 10 mM Tris-HCl (pH 7.8), 4% glycerol, 1 mM MgCl_2_, and 1 mg of poly dI-dC as nonspecific competitors, in a final reaction volume of 20 **μ**l. The reactions were run on a 5% polyacrylamide gel, in 0.5x Tris-borate-EDTA buffer to visualize complex formation by retardation of the ^32^P-labeled target DNA. In some experiments monoclonal anti-Abd-B or anti-GFP were incubated with aliquots of the reaction mixture for an additional 30 min. For each gel shift reaction, a control with cell extract of non-trans00FEcted cells was used to detect possible DNA binding by endogenous lysate factors. Gels were dried at 80°C in vacuum, exposed to a phosphorimager screen and detected by a typhoon scanner.

## Supporting Information

S1 Fig JAK/STAT upregulates *crb* expression in the posterior spiracles. (A, B) Higher levels of Crb protein are expressed in the posterior spiracles and tracheae primordia than in the surrounding embryonic ectoderm. Inset in (A) shows an A8 close up. (C) *upd* RNA expression. (D, E) Posterior spiracle specific **β**-Gal expression driven by the *crb43*.*2* CRM. (F) Mutation of two STAT binding sites in *crb43*.*2* decreases posterior spiracle reporter expression. (G-G”) Cells with activated JAK/STAT signalling express the 10xSTAT-dGFP reporter in both posterior spiracles and trachea primordia (green in G and grey in G’). Ct staining (red in G and grey in G”) labels the internal posterior spiracle cells. Arrowheads: posterior spiracle primordium; arrows: tracheal pits. Ml: Mandibulary, Mx: Maxillary, Lb: Labial, T1-T3: Thoracic and A1-A9: Abdominal segments.
(TIF)

S2 Fig Characterization of the *crb* posterior spiracle JAK/STAT regulated minimal CRM. (A) Chromatin Immunoprecipitation (ChIP) shows activated STAT92E-GFP binds to the *crb* CRM in S2 cells. Cells were transfected with pAC-Gal4 to activate the expression of UAS constructs, pAC-Hop^Tum-l^ expressing an activated JAK kinase to induce STAT92E activation by phosphorylation and either with pUAST-STAT92E-GFP or pUAST alone as a control. ChIP of STAT92E was performed using an anti-GFP antibody. (B) Sequence conservation of the *crb43*.*2* across the aligned genomes of twelve *Drosophila* species together with mosquito (*A*. *gambiae*), honeybee (*A*. *mellifera*) and beetle (*T*. *casteneum*). *crb518* location is highlighted in red. Asterisks indicate the 4N STAT92E binding sites. (C) **β**-Gal expression driven by *crb518*. (D) Expression of *crb518* in *Abd-B* ^*M1*^ mutant embryos. (E) Expression of *crb518* in *Df(1)os1A* mutant embryos lacking the three Unpaired ligands. (F) Expression of *crb518* when both STAT92E sites are mutated (*crb518-STAT-mt-Z*). Arrowheads; posterior spiracle primordium.
(TIF)

S3 Fig The spiracle specificity module does not contain cryptic STAT92E binding sites. (A) Localization of the putative cryptic STAT92E binding sites in *crb305* deviating by one (orange) or two nucleotides (blue) from the consensus (S) and oligos used in EMSAs to determine binding of activated STAT92E-GFP. Oligos with mutated STAT consensus sites (x) were used as negative controls. (B) EMSAs showing that only STAT consensus sites are bound by activated STAT92E as shown by the band shifts (black arrows) and by supershifts (grey arrows) in the presence of anti-GFP (lanes 1–5, 11–15, 36–40, 56–60); neither mutated STAT sites (lanes 6–10, 16–20) nor the putative cryptic sites (lanes 21–35, 41–55, 61–80) exhibited binding by activated STAT92E-GFP. As previously reported [14], addition of the antibody in the supershift stabilizes STAT92E binding to DNA.
(TIF)

S4 Fig Localization of the repressor element of *crb518*. (A) Analysis of the repressor module sequence conservation across the aligned genomes of twelve *Drosophila* species together with the mosquito (*A*. *gambiae*), the honeybee (*A*. *mellifera*) and the beetle (*T*. *casteneum*), shows two highly conserved elements CE1 (345–385 bp) and CE2 (492–518 bp) intercalated by two non-conserved elements NC1 (306–344 bp) and NC2 (386–491 bp). The expression of *crb305-STAT-mt* reporter (B) is not affected by the addition of NC1 (C), or NC1 and CE1 (D), however, the addition of NC1, CE1 and NC2 downregulates expression in the posterior spiracles (E). Arrowheads: posterior spiracles primordium.
(TIF)

S5 Fig Ectopic *upd* induces ectopic expression of *crb305* in the posterior abdominal segments. (A) Wild type expression of crb305 reporter (green in A and A'') is mostly restricted to the posterior spiracles as shown by co-expression of the posterior spiracle marker Ct (red in A and A'). (B, B”) In *69B-Gal4 UAS-upd* embryos *crb305* is only expressed ectopically in the posterior abdominal segments, despite *69B-Gal4* driving expression in the ectoderm of more anterior segments. Note that while *crb305* (green) is ectopically activated, Ct (red) is maintained to the posterior spiracle anlage (B, B’). Images show confocal stainings of st12 embryos. Arrows: posterior spiracles primordium.
(TIF)

S6 Fig Expression and function of the [CH321-91P18] BAC-*Abd-B-gfp* construct. (A) *BAC-Abd-B-gfp* embryo double stained with anti-Abd-B (A, A’, red) and anti-GFP (A, A”, green) at st14. Abd-B-GFP expression (A'') is restricted to PS13-PS14 and only the endogenous Abd-B protein is detected in PS10-12 (red in A and A'). (B) Ventral view of a late wild type embryo cuticle showing the large denticle belts present in the abdominal segments compared with the smaller thoracic denticle belts. (B') shows a dorsal view of the same cuticle focusing on the posterior spiracles. (C) Ventral view of a *Ubx* ^*MX12*^, *abd-A* ^*M1*^, *Abd-B* ^*M8*^ triple mutant embryo cuticle showing the abdominal denticle belts have the same aspect as the T2 thoracic belts. (C') shows a dorsal view of the same cuticle where the posterior spiracles are missing. (D) Ventral view of the cuticle of a *Ubx* ^*MX12*^, *abd-A* ^*M1*^, *Abd-B* ^*M8*^ triple mutant embryo heterozygous for BAC-*Abd-B-gfp* showing little rescue in the abdominal denticle belts which almost have the same aspect as the T2 thoracic belts. (D') dorsal view of the same cuticle showing the posterior spiracles are partially rescued with the formation of small filzkörpers. In A-A”, arrow indicates the posterior spiracle and the arrowhead, the trachea; in B’, C’ and D’, arrows indicate the position of the posterior spiracles.
(TIF)

S1 Table Primers used to generate the different reporter constructs. (XLSX)

S2 Table Primers used to generate double stranded oligonucleotides used in the EMSAs with STAT92E-GFP. An additional GGA and GAG (in bold) was added to the 5’ end of the forward and reverse primers, respectively, to allow labelling with [**α**-^32^P]dCTP.
(XLSX)

S3 Table Primers used to generate double stranded oligonucleotides used in the EMSAs with Abd-Bm-HA. An additional GGA and GAG (in bold) was added to the 5’ end of the forward and reverse primers, respectively, to allow labelling with [**α**-^32^P]dCTP.
(XLSX)
