# Supplementary figures and images for: JAK/STAT and Hox Dynamic Interactions in an Organogenetic Gene Cascade
Source: PLoS Genet. 2015 Jul 31;11(7):e1005412. doi: 10.1371/journal.pgen.1005412 (PMC4521708; doi:10.1371/journal.pgen.1005412)

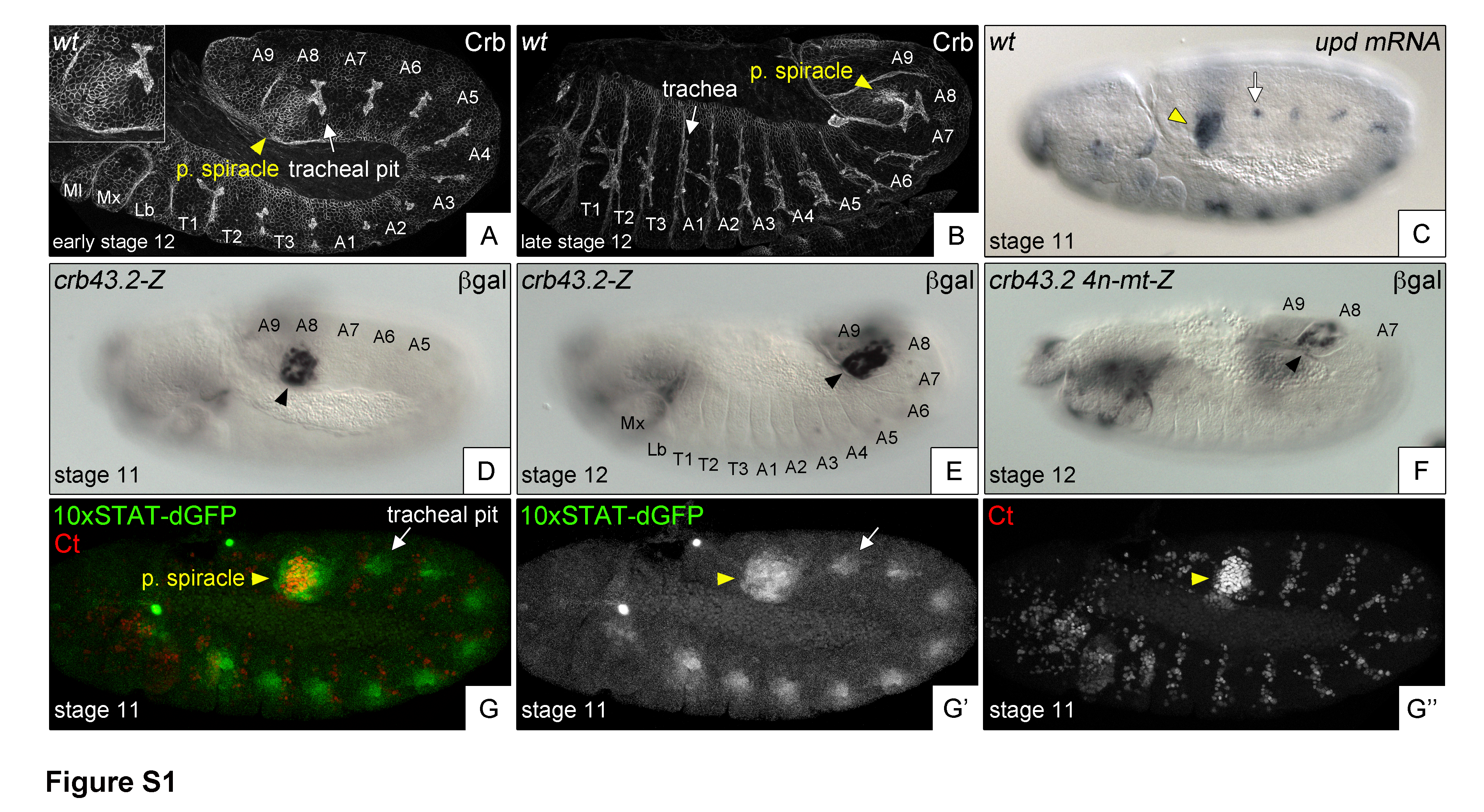

Supplement: S1 Fig — (A, B) Higher levels of Crb protein are expressed in the posterior spiracles and tracheae primordia than in the surrounding embryonic ectoderm. Inset in (A) shows an A8 close up. (C) upd RNA expression. (D, E) Posterior spiracle specific β-Gal expression driven by the crb43.2 CRM. (F) Mutation of two STAT binding sites in crb43.2 decreases posterior spiracle reporter expression. (G-G”) Cells with activated JAK/STAT signalling express the 10xSTAT-dGFP reporter in both posterior spiracles and trachea primordia (green in G and grey in G’). Ct staining (red in G and grey in G”) labels the internal posterior spiracle cells. Arrowheads: posterior spiracle primordium; arrows: tracheal pits. Ml: Mandibulary, Mx: Maxillary, Lb: Labial, T1-T3: Thoracic and A1-A9: Abdominal segments. (TIF) [file pgen.1005412.s001.tif]

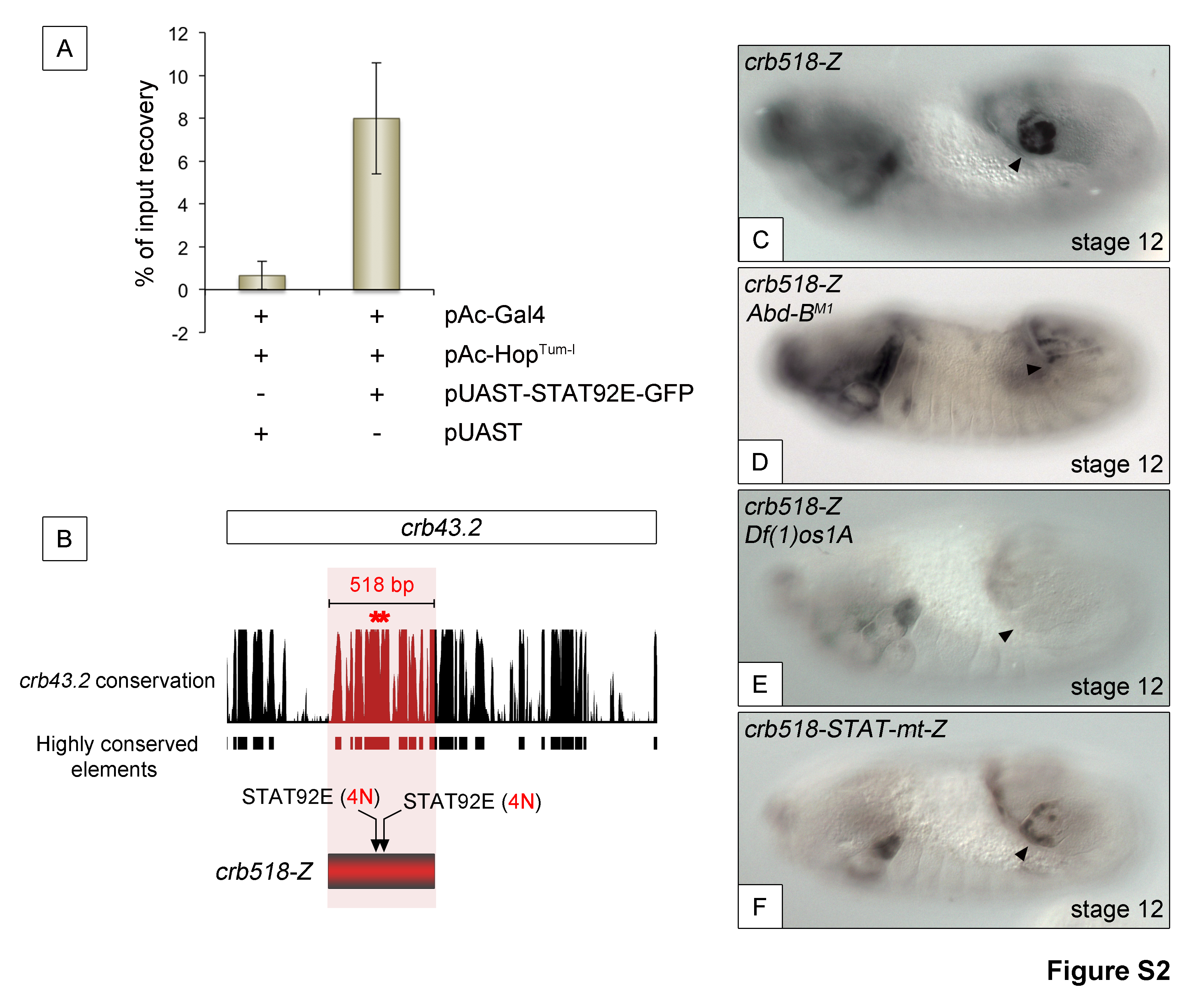

Supplement: S2 Fig — (A) Chromatin Immunoprecipitation (ChIP) shows activated STAT92E-GFP binds to the crb CRM in S2 cells. Cells were transfected with pAC-Gal4 to activate the expression of UAS constructs, pAC-HopTum-l expressing an activated JAK kinase to induce STAT92E activation by phosphorylation and either with pUAST-STAT92E-GFP or pUAST alone as a control. ChIP of STAT92E was performed using an anti-GFP antibody. (B) Sequence conservation of the crb43.2 across the aligned genomes of twelve Drosophila species together with mosquito (A. gambiae), honeybee (A. mellifera) and beetle (T. casteneum). crb518 location is highlighted in red. Asterisks indicate the 4N STAT92E binding sites. (C) β-Gal expression driven by crb518. (D) Expression of crb518 in Abd-B M1 mutant embryos. (E) Expression of crb518 in Df(1)os1A mutant embryos lacking the three Unpaired ligands. (F) Expression of crb518 when both STAT92E sites are mutated (crb518-STAT-mt-Z). Arrowheads; posterior spiracle primordium. (TIF) [file pgen.1005412.s002.tif]

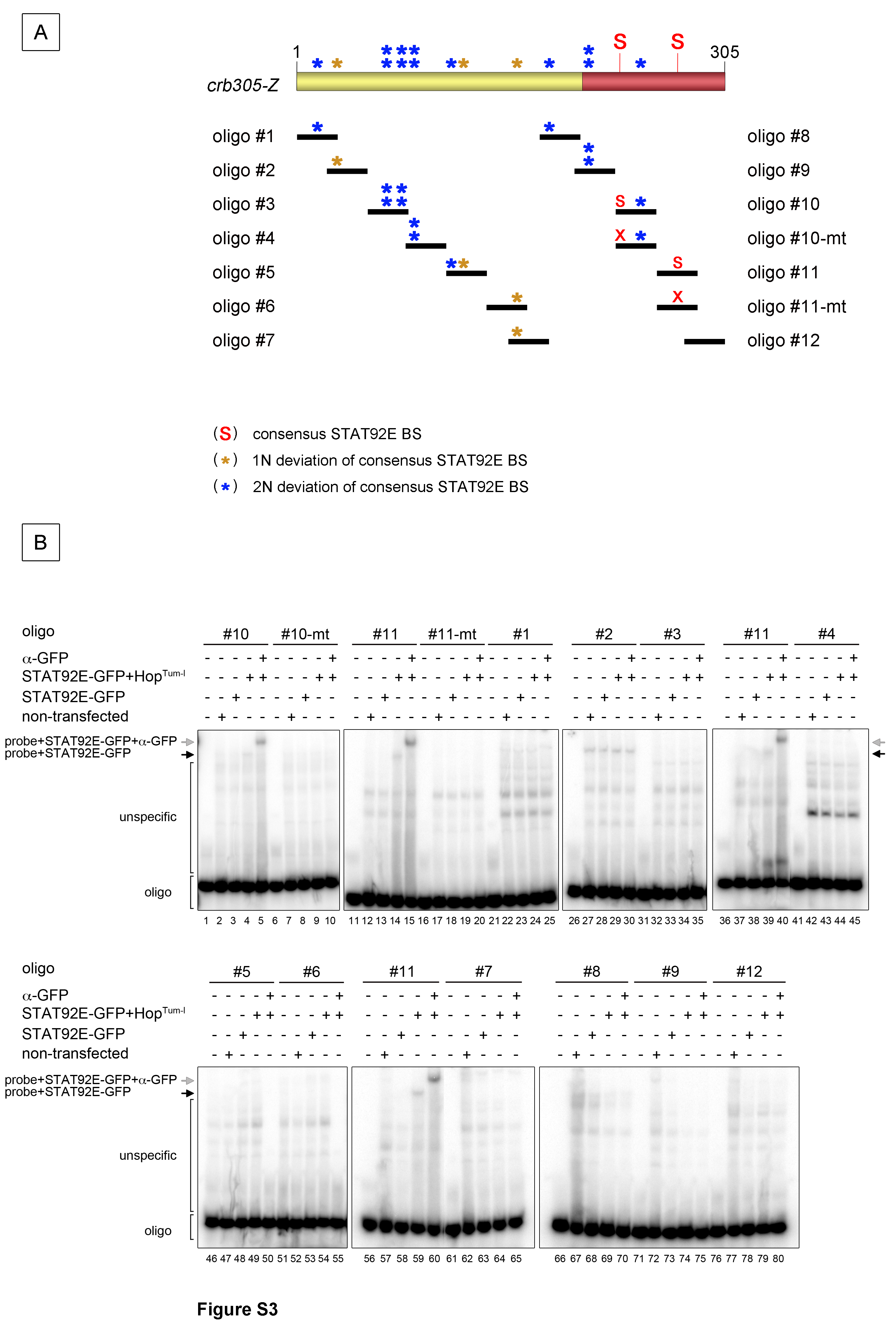

Supplement: S3 Fig — (A) Localization of the putative cryptic STAT92E binding sites in crb305 deviating by one (orange) or two nucleotides (blue) from the consensus (S) and oligos used in EMSAs to determine binding of activated STAT92E-GFP. Oligos with mutated STAT consensus sites (x) were used as negative controls. (B) EMSAs showing that only STAT consensus sites are bound by activated STAT92E as shown by the band shifts (black arrows) and by supershifts (grey arrows) in the presence of anti-GFP (lanes 1–5, 11–15, 36–40, 56–60); neither mutated STAT sites (lanes 6–10, 16–20) nor the putative cryptic sites (lanes 21–35, 41–55, 61–80) exhibited binding by activated STAT92E-GFP. As previously reported [14], addition of the antibody in the supershift stabilizes STAT92E binding to DNA. (TIF) [file pgen.1005412.s003.tif]

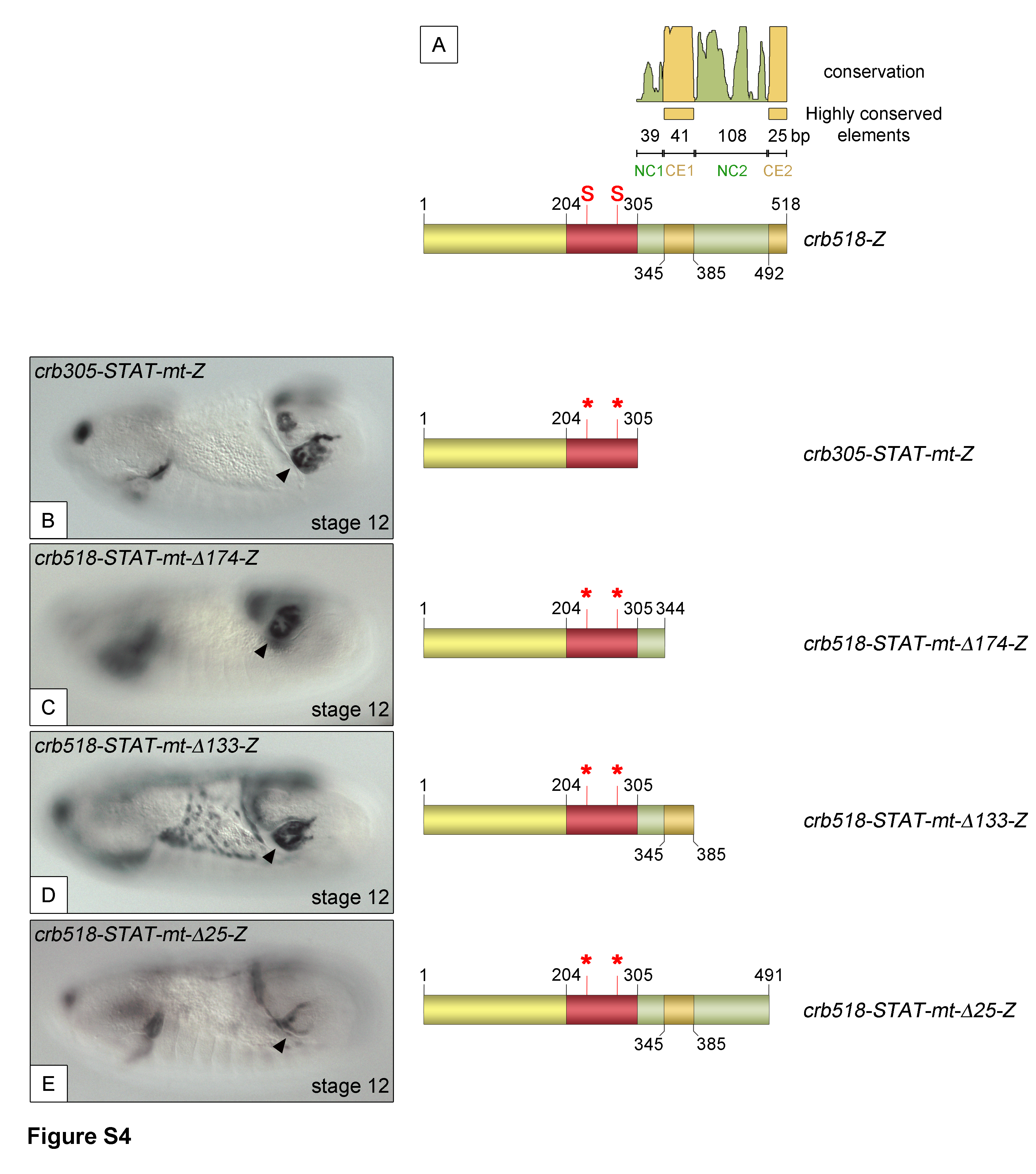

Supplement: S4 Fig — (A) Analysis of the repressor module sequence conservation across the aligned genomes of twelve Drosophila species together with the mosquito (A. gambiae), the honeybee (A. mellifera) and the beetle (T. casteneum), shows two highly conserved elements CE1 (345–385 bp) and CE2 (492–518 bp) intercalated by two non-conserved elements NC1 (306–344 bp) and NC2 (386–491 bp). The expression of crb305-STAT-mt reporter (B) is not affected by the addition of NC1 (C), or NC1 and CE1 (D), however, the addition of NC1, CE1 and NC2 downregulates expression in the posterior spiracles (E). Arrowheads: posterior spiracles primordium. (TIF) [file pgen.1005412.s004.tif]

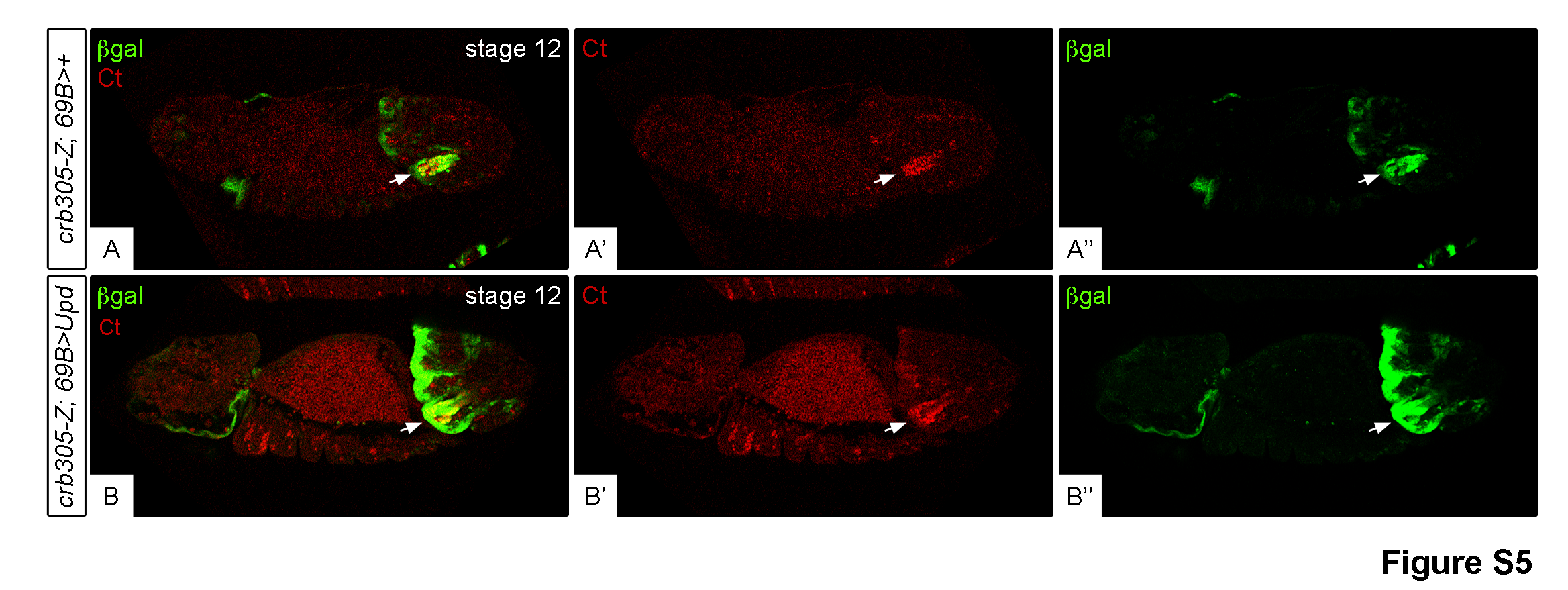

Supplement: S5 Fig — (A) Wild type expression of crb305 reporter (green in A and A'') is mostly restricted to the posterior spiracles as shown by co-expression of the posterior spiracle marker Ct (red in A and A'). (B, B”) In 69B-Gal4 UAS-upd embryos crb305 is only expressed ectopically in the posterior abdominal segments, despite 69B-Gal4 driving expression in the ectoderm of more anterior segments. Note that while crb305 (green) is ectopically activated, Ct (red) is maintained to the posterior spiracle anlage (B, B’). Images show confocal stainings of st12 embryos. Arrows: posterior spiracles primordium. (TIF) [file pgen.1005412.s005.tif]

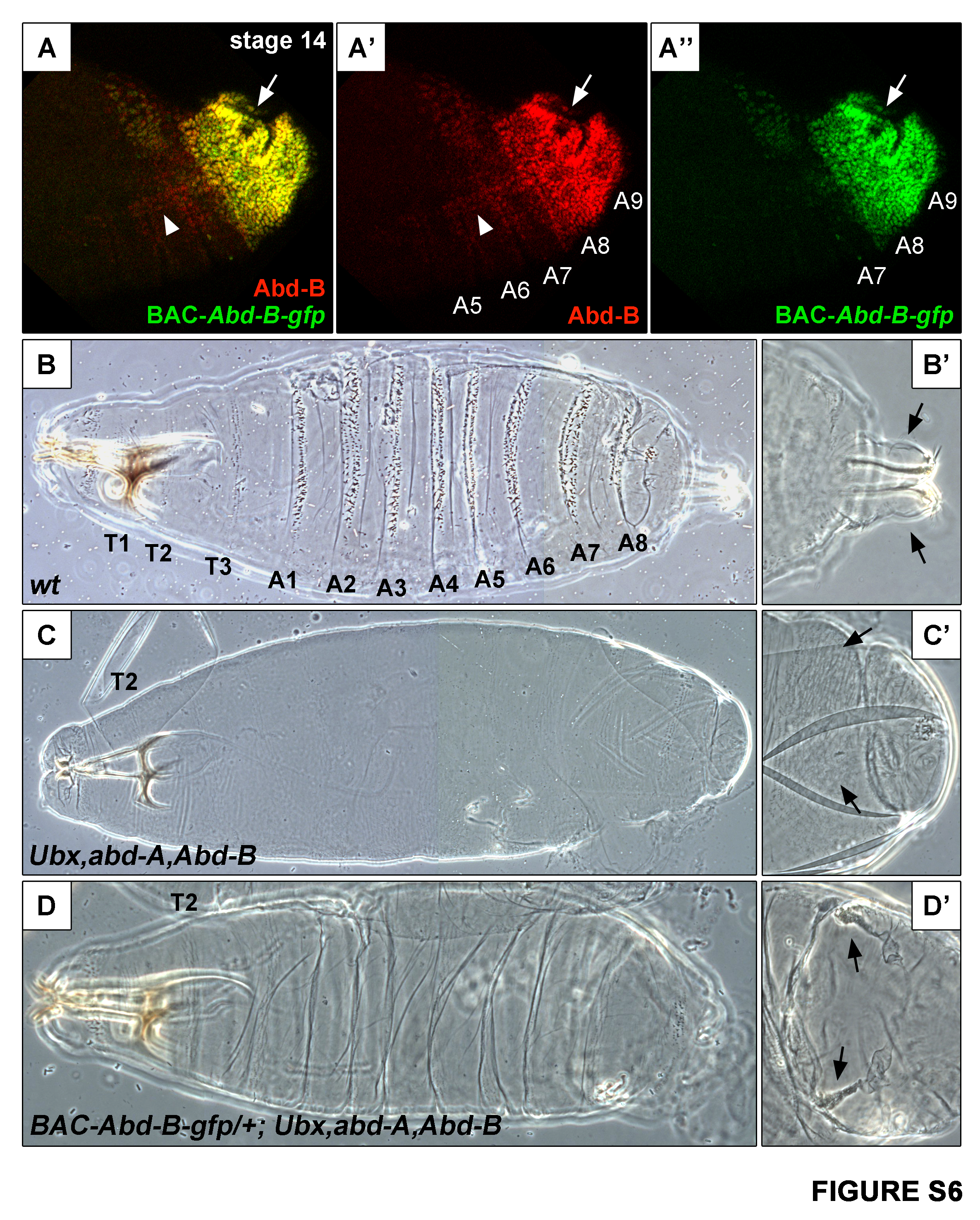

Supplement: S6 Fig — (A) BAC-Abd-B-gfp embryo double stained with anti-Abd-B (A, A’, red) and anti-GFP (A, A”, green) at st14. Abd-B-GFP expression (A'') is restricted to PS13-PS14 and only the endogenous Abd-B protein is detected in PS10-12 (red in A and A'). (B) Ventral view of a late wild type embryo cuticle showing the large denticle belts present in the abdominal segments compared with the smaller thoracic denticle belts. (B') shows a dorsal view of the same cuticle focusing on the posterior spiracles. (C) Ventral view of a Ubx MX12, abd-A M1, Abd-B M8 triple mutant embryo cuticle showing the abdominal denticle belts have the same aspect as the T2 thoracic belts. (C') shows a dorsal view of the same cuticle where the posterior spiracles are missing. (D) Ventral view of the cuticle of a Ubx MX12, abd-A M1, Abd-B M8 triple mutant embryo heterozygous for BAC-Abd-B-gfp showing little rescue in the abdominal denticle belts which almost have the same aspect as the T2 thoracic belts. (D') dorsal view of the same cuticle showing the posterior spiracles are partially rescued with the formation of small filzkörpers. In A-A”, arrow indicates the posterior spiracle and the arrowhead, the trachea; in B’, C’ and D’, arrows indicate the position of the posterior spiracles. (TIF) [file pgen.1005412.s006.tif]
